# Modulation of neural networks and symptom correlated in fibromyalgia: A randomized double-blind multi-group explanatory clinical trial of home-based transcranial direct current stimulation

**DOI:** 10.1371/journal.pone.0288830

**Published:** 2024-11-13

**Authors:** Rael Lopes Alves, Maxciel Zortea, Paul Vicuña Serrano, Vani dos Santos Laranjeira, Betina Franceschini Tocchetto, Leticia Ramalho, Camila Fernanda da Silveira Alves, Rafaela Brugnera Tomedi, Rodrigo Pereira de Almeida, Samara Machado Bruck, Liciane Medeiros, Paulo R. S. Sanches, Danton P. Silva, Iraci L. S. Torres, Felipe Fregni, Wolnei Caumo

**Affiliations:** 1 Post-Graduate Program in Medical Sciences, School of Medicine, Universidade Federal do Rio Grande do Sul, Porto Alegre, Brazil; 2 Laboratory of Pain and Neuromodulation, Hospital de Clínicas de Porto Alegre, Porto Alegre, Brazil; 3 Health School, University of Vale do Rio dos Sinos, São Leopoldo, Porto Alegre, Brazil; 4 Post-Graduate Program in Health and Human Development, Universidade La Salle, Canoas, Brazil; 5 Laboratory of Biomedical Engineer, Hospital de Clínicas de Porto Alegre, Porto Alegre, Brazil; 6 Pharmacology of Pain and Neuromodulation: Pre-Clinical Investigations Research Group, Universidade Federal Do Rio Grande Do Sul, Porto Alegre, Brazil; 7 Laboratory of Neuromodulation and Center for Clinical Research Learning, Physics and Rehabilitation Department, Spaulding Rehabilitation Hospital, Boston, Massachusetts, United States of America; 8 Pain and Palliative Care Service, Hospital de Clínicas de Porto Alegre, Porto Alegre, Brazil; 9 Department of Surgery, School of Medicine, Universidade Federal Do Rio Grande Do Sul, Porto Alegre, Brazil; UFSCar: Universidade Federal de Sao Carlos, BRAZIL

## Abstract

**Background:**

Transcranial direct current stimulation (tDCS) might modulate neural activity and promote neural plasticity in patients with fibromyalgia (FM). This multi-group randomized clinical trial compared home-based active tDCS (HB-a-tDCS) on the left dorsolateral prefrontal cortex (l-DLPFC) or home-based sham tDCS (HB-s-tDCS), and HB-a-tDCS or HB-s-tDCS on the primary motor cortex (M1) in the connectivity analyses in eight regions of interest (ROIs) across eight resting-state electroencephalography (EEG) frequencies.

**Methods:**

We included 48 women with FM, aged 30 to 65, randomly assigned to 2:1:2:1 to receive 20 sessions during 20 minutes of HB-a-tDCS 2mA or HB-s-tDCS, over l-DLPFC or M1, respectively. EEG recordings were obtained before and after treatment with eyes open (EO) and eyes closed (EC).

**Results:**

In the EC condition, comparing pre to post-treatment, the HB-a-tDCS on l-DLPFC decreased the lagged coherence connectivity in the delta frequency band between the right insula and left anterior cingulate cortex (ACC) (t = -3.542, p = .048). The l-DLPFC HB-a-tDCS compared to HB-s-tDCS decreased the lagged coherence connectivity in the delta frequency band between the right insula and left ACC (t = -4.000, p = .017). In the EO condition, the l-DLPFC HB-a-tDCS compared to M1 HB-s-tDCS increased the lagged coherence connectivity between the l-DLPFC and left ACC in the theta band (t = -4.059, p = .048). Regression analysis demonstrated that the HB-a-tDCS effect on the l-DLPFC was positively correlated with sleep quality. On the other hand, the HB-a-tDCS on l-DLPFC and HB-s-tDCS on M1 were positively correlated with pain catastrophizing.

**Conclusions:**

These results show that HB-a-tDCS affects the neural connectivity between parts of the brain that control pain’s emotional and attentional aspects, which are most noticeable at lower EEG frequencies in a rest state. This effect on neural oscillations could serve as a neural marker associated with its efficacy in alleviating fibromyalgia symptoms.

**Clinical trial registration:**

identifier [NCT03843203].

## 1. Introduction

Fibromyalgia (FM) is a primary chronic pain condition classified as nociplastic pain due to the absence of a straightforward pathophysiological process and intense emotional distress [1]. Its symptoms include musculoskeletal pain, fatigue, non-restorative sleep, cognitive changes, depressive symptoms, and other correlates of autonomic dysfunction, such as irritable bowel syndrome and bladder tenesmus [2]. The exact mechanism underlying this syndrome is not fully understood, but increased facilitatory modulation and dysfunctional descending inhibitory pathway activity may contribute to central sensitization [3]. Neuroimaging studies provide further insight into the underlying central nervous system (CNS) changes in central sensitization, demonstrating significant structural, chemical, and functional alterations in pain-processing regions such as the thalamus, periaqueductal gray (PAG), insula, cingulate, and somatosensory cortices [4, 5].

The neural network underlying the pain experience emphasizes the dynamics of functional connectivity (FC) that underpin the pain experience [6]. FC is the degree to which activity between a pair of brain regions covaries or correlates over time. In FM, the descending pain modulatory system (DPMS) does not work as well when there is more FC between pain processing areas like the left motor cortex (MC) and the left prefrontal cortex (PFC), as well as between the left MC and the right PFC [7]. Another study in FM found an increased delta value of FC in response to acute pain between the PFC and MC in genotypes of brain-derived neurotrophic factor (BDNF) Val/Met compared to Val/Val [8]. In contrast, the Val/Val group showed decreased interhemispheric connectivity in these areas, less efficiency of inhibitory DPMS, and a higher impact of fibromyalgia symptoms on quality of life [8]. An analysis of frequency maps using electroencephalography (EEG) in FM revealed anomalous FC at the bilateral precuneus, with right predominance, on the right inferior parietal cortex, bilateral prefrontal cortex, medial cortex, and right anterior cingulate cortex (ACC) [9]. Coherence analysis of the brain signal showed apparent differences between FM and controls, particularly in the bilateral frontotemporal region. Discriminatory analysis indicated a significant difference in group interconnectivity patterns [9].

In a recent study, we found heightened coherence connectivity among various neural circuits involved in processing pain information in individuals with FM compared to healthy controls. The study revealed increased connectivity between the left dorsolateral prefrontal cortex (DLPFC) and right ACC, as well as between the left insula and right DLPFC, particularly in the beta-3 frequency band. This heightened connectivity was associated with the neuroplasticity state, as evidenced by levels of BDNF in the serum, and correlated with the severity of FM symptoms [10].

Even though there is evidence that dysfunctional processing in FM is caused by dysfunctional connectivity and dysfunction of the DPMS [7, 11], there is not much evidence about how treatment affects these neural markers. Thus, it is crucial to investigate the effect of treatments on these neural markers associated with the severity of pain symptoms, which can help develop effective treatments for pain management and personalized treatment. Such neural features, including cerebral oscillations and connectivity in response to treatment, can provide insights into the mechanisms of action of the treatment and whether its efficacy in clinical outcomes is related to changes in these neural markers. This way, personalized therapies based on each patient’s neuroplasticity state can be tailored. Following this rationale, it is reasonable to investigate the effect of neuromodulatory therapies that counter-regulate dysfunctional neuroplasticity underpinning chronic pain. Among these therapies is transcranial direct current stimulation (tDCS), which modulates neural cortical and subcortical networks. According to prior studies in FM, long-term tDCS at home (sixty sessions) effectively reduced analgesic use by 55% and reduced pain scores [12]. Along the same line, other trials with FM found that four weeks of home-based tDCS (HB-tDCS) improved pain-related catastrophizing [13] and cognitive impairment [14]. The evidence supporting the effectiveness of tDCS in treating FM is increasing. In line with this, a study on long-term HB-tDCS consisting of sixty sessions showed a significant reduction in pain scores and analgesic use by 55% [12]. Meta-analyses and systematic reviews consistently demonstrate the positive impact of tDCS on FM symptoms, with tDCS over the primary motor cortex (M1) showing greater efficacy in reducing pain scores compared to DLPFC stimulation [15, 16]. Also, evidence-based guidelines recommend using tDCS in FM for its benefits in pain management [17, 18]. Overall, tDCS over M1 enhanced pain pressure thresholds, and improved quality of life in FM patients. Conversely, DLPFC stimulation has improved fatigue levels [19] and pain catastrophizing [13]. These findings suggest that the analgesic effects of tDCS vary depending on the target stimulation, with M1 stimulation being more effective in alleviating FM pain symptoms [20].

These set data suggest that the top-down effect of tDCS on clinical outcomes may depend on the area that is stimulated and the type of current (anodal or cathodal). The M1 modulates neural networks associated with the somatosensory system through cortical effects in the thalamic nucleus, ACC, and brainstem [21]. Anodal tDCS on M1 induces corticothalamic inhibition of the ventral posterolateral nucleus (VPL), which is responsible for discriminatory sensitivity, and the ventral posteromedial nucleus (VPM), which is responsible for nociceptive sensation. Stimulation of the DLPFC decreases the activity of the midbrain-medial thalamic pathway, which is involved in the modulation of structures related to the emotional perception of pain [21]. Thus, the effect of tDCS depends on the site to be stimulated (DLPFC or M1) and the type of stimulation (anodic or cathodic) and is likely related to the number of stimulation sessions. Even though there is growing evidence of the clinical benefits of tDCS, there needs to be more literature regarding its neurophysiological effects on areas involved in pain processing and oscillatory brain signatures. Thus, additional studies are required to better understand the mechanisms of tDCS as a treatment for pain and correlated symptoms.

The study aimed to evaluate the impact of self-administered home-based active-tDCS (HB-a-tDCS) on the functional connectivity between pain-related areas in the brain. Specifically, it focused on the effects of HB-a-tDCS on the left DLPFC and the M1 compared to home-based sham-tDCS (HB-s-tDCS). The study sought to determine whether HB-a-tDCS would enhance lagged coherence connectivity in the resting-state EEG more than HB-s-tDCS and how this enhancement is linked to clinical measures related to pain and serum BDNF levels. The hypothesis was that active treatments would be more effective than sham in improving brain lagged coherence connectivity in key pain processing areas, potentially leading to improvements in clinical pain measures.

## 2. Materials and methods

### 2.1 Study design and eligibility

The trial’s protocol for the Certificate of Presentation for Ethical Appreciation (CAAE) registry number is 36995020.3.0000.5327, and the study was approved by the Research Ethics Committee at the Hospital de Clínicas de Porto Alegre (HCPA), Brazil, under registration in the Institutional Review Board (IRB) number 2020–0369. Data collected were achieved in the outpatient departments of the clinical research center of HCPA and in the research rooms of the laboratory of pain and neuromodulation of HCPA. Informed consent was obtained from all participating patients, who provided verbal and written consent to participate in the trial. The study followed a randomized, double-blind, and sham-controlled design, ensuring that neither the patients nor the researchers involved knew the treatment assignment. It is important to note that the participants in the trial did not receive any form of compensation for their involvement.

The study started in September 2019 but was paused in March 2020 due to the COVID-19 pandemic. However, in November 2020, the study resumed with several modifications and restrictions to prioritize the safety of participants and researchers. Data collection was completed in November 2022. The collected data were available on the public repository figshare DOI: https://doi.org/10.6084/m9.figshare.23542116.v1.

We adjusted the study procedures to address the challenges posed by the pandemic. Specific hospital visit dates were scheduled, and the duration of visits for psychophysical measures and device-related activities was limited. Initially, the protocol required formal consent and face-to-face assessments to gather information on clinical symptoms and demographic characteristics. However, in response to the pandemic, online methods were implemented for information collection, prioritizing the health and well-being of all involved in the study. Participants who had contracted the virus before the data collection phase was temporarily paused were placed on hold until they received a negative COVID-19 test result.

Once they tested negative, they underwent an evaluation to determine their eligibility for inclusion in the study. These adaptations were implemented to ensure the research could continue without interruption while adhering to the necessary safety measures during the pandemic.

### 2.2 Inclusion and exclusion criteria

We included in the study women aged 30 to 65, literate, and diagnosed with FM according to the American College of Rheumatology (ACR) 2016 [22]. Participants needed to report a score of 6 or higher on the Numerical Pain Scale (NPS 0–10) [23, 24] most of the time in the last three months. Subjects were excluded in cases of pregnancy, if they had a neurological disease, a history of head trauma or neurosurgery, or a history of alcohol or drug abuse in the last six months. Besides, participants could not have decompensated systemic diseases, chronic inflammatory diseases, uncompensated hypothyroidism, another metabolic disease, or be getting treatment for cancer.

**Fig 1:**
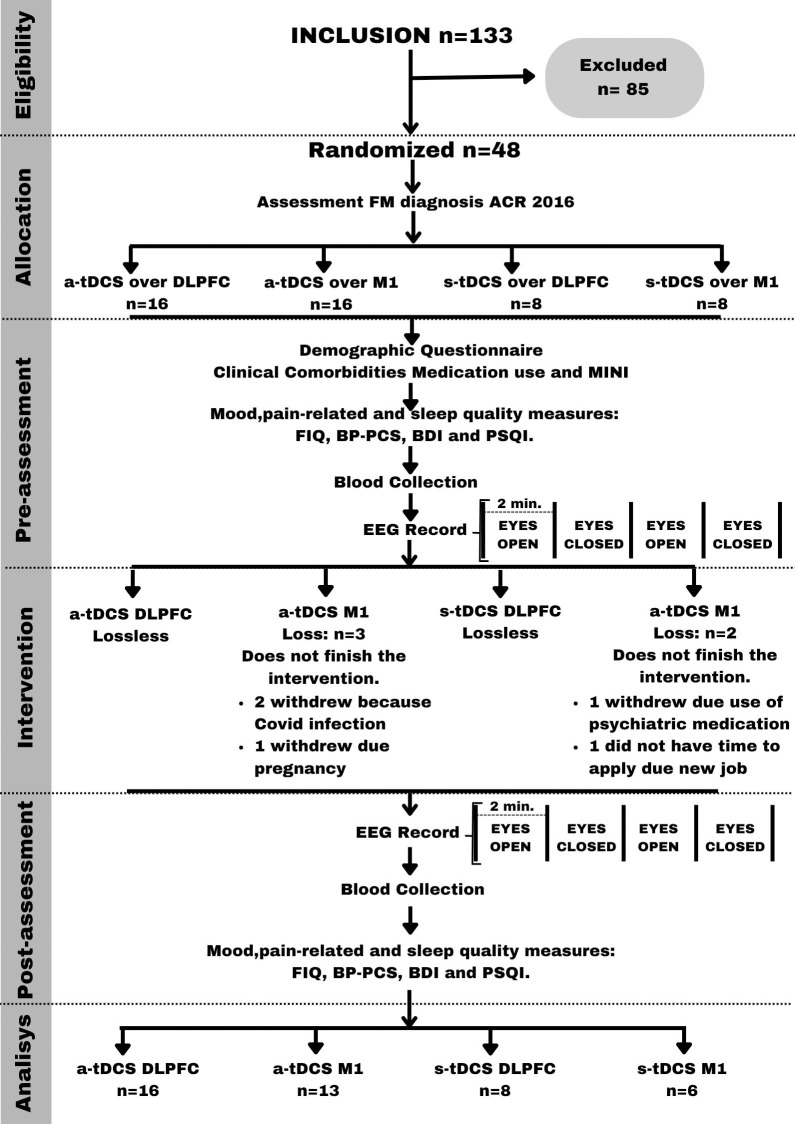
Flowchart of the study assessments. FM = Fibromyalgia; ACR = American College of Rheumatology; FIQ = Fibromyalgia Impact Questionnaire; BDI = Beck Depression Inventory; MINI = Mini International Neuropsychiatric Interview; BP-PCS = Pain Catastrophizing Scale; BP-CSI = Central sensitization inventory—Brazilian Portuguese version; PSQI = Pittsburgh Sleep Quality Index; EEG = Electroencephalography; a-tDCS = active transcranial direct current stimulation; s-tDCS = sham transcranial direct current stimulation; DLPFC = Dorsolateral prefrontal cortex; M1 = Primary Motor cortex.

We screened 133 FM participants eligible to participate in this study. However, 85 were excluded for different reasons, such as living far away from the research center, having trouble getting around on public transportation, being unemployed, etc. Some of the screened participants did not fulfill the diagnostic criteria for FM. Besides, they were excluded if their pain levels were lower than 6 (NPS 0–10) or they had another uncompensated clinical disease (rheumatoid arthritis, lupus, hypothyroidism, etc.). So, 48 FM were included in the study, but 5 of them were excluded. The modified intention-to-treat analysis (mITT) included subjects completing ≥ 50% of sessions (10 sessions). So, at the end, 43 subjects were included in the analysis. The sequence of screening and assessments is presented in Fig 1.

### 2.3 Intervention

Participants received the programmed HB-tDCS device. The treatment was performed during weekdays over 4 weeks, totaling 20 sessions. Three devices were misprogrammed and exceeded the pre-defined number of sessions. We decided not to withdraw these data from the analysis because they did not represent significant differences in the number of sessions per group. The current applied was 2 mA for 20 minutes to the active stimulation delivered using the anode electrode positioned over the left M1 and cathodal stimulation over the contra-lateral supra-orbital area for the first assembly. In the second montage, anodal stimulation was placed over the left DLPFC and cathodal stimulation over the right DLPFC. The HB-a-tDCS condition is composed of a ramp-up time of 30 s for the current to go from zero to 2 mA and a ramp-down time that also takes 30 s for the current to go from 2 mA to zero to end the stimulation. For HB-s-tDCS conditions, the montage was the same as HB-a-tDCS. A 30-s ramp-up in intensity from zero to 2 mA was used for HB-a-tDCS and HB-s-tDCS, as well as a ramp-down for about the same duration, as explained in the blinding session. To improve the current supplied was used two silicone cannulas were attached to 35 cm2 (5x7 cm) electrodes coated in sponges wet with saline solution. The HB-tDCS safety was evaluated using a questionnaire based on previously reported adverse events. Details about the protocol can be seen in S2 File Checklist to use tDCS at home protocol and in the HB-tDCS updated protocol [25].

### 2.4 Outcomes and instruments of assessments

The primary outcome was the lagged coherence connectivity in different EEG frequency bands (delta, theta, alpha-1, alpha-2, beta-1, beta-2, beta-3, and gamma) at resting state. These measurements were taken between regions of interest (ROIs) in the pain network. The assessments were conducted in a specific sequence, as shown in Fig 1.

#### 2.4.1 Assessment of primary outcome

The electroencephalography was recorded using 18 scalp sites according to the 10–20 system, FP1, FP2, F7, F3, Fz, F4, F8, T7, C3, Cz, C4, T8, P7, P3, Pz, P4, P8, Oz, and the left ear (EXT), with reference to the right ear (CMS/DRL). The procedures were conducted with subjects sitting in a comfortable armchair, in a quiet place, and with the light of the room off. The EEG system used was the ENOBIO 20, Neuroelectrics (Barcelona, SP), which comprises an EEG cap with circular gel electrodes with a contact area of 1.75 cm^2^. Impedance was 5 kΩ for all electrodes, with high dynamic resolution (24 bits, 0.05uV) and a sampling rate of 500 Hz. A line noise filter (60-Hz) was applied to remove main line artifacts from the EEG data.

We used the EEG resting-state paradigm collected for 8 minutes total, with 2 minutes switched between EC and EO conditions. Participants were instructed to remain awake, relaxed, and thinking-free. During the EO condition, participants were instructed to fixate their vision on a small cross presented in front of them. The EC condition is the level of arousal at rest, and the EO condition is the level of arousal at activation [26, 27]. The supplementary material S4 File provides detailed information about the EEG resting-state protocol.

#### 2.4.2 Preprocessing and functional connectivity analysis

The EEG data preprocessing was conducted with the open-source toolbox EEGLAB 14.1 [28], which ran in the MATLAB environment (The MathWorks Inc., Natick, Massachusetts, United States). Artifact detection was performed through visual inspection, removing segments of bad channels, if necessary. Continuous EEG data was band-pass filtered using a simple FIR filter with cutoff frequencies of 0.5–40 Hz, resampled to 250 Hz, and split into 4.096 s epochs.

Epochs containing artifacts were automatically excluded from the analysis. Rejection thresholds were fixed according to artifact characteristics from non-filtered continuous EEG as following [29]:

a) 50μV thresholds were set for FP1 and FP2 electrodes and 100μV thresholds for other electrodes to eliminate eye blinks and other quick movements from non-filtered continuous EEG were eliminated,

b) 50μV thresholds were used for slow waves (0–1 Hz band), while 30μV thresholds were used for fast waves (20–35 Hz band) to eliminate artifacts associated with slow head or body movements,

Brain connectivity was computed establishing the minimum threshold to be 40 seconds for each resting-state condition (EO, EC) [30]. Subjects or conditions below this threshold was rejected for the analysis.

Standardized low-resolution brain electromagnetic tomography (sLORETA) was used to measure the functional connectivity, which compute the linear dependence (coherence) of the electric neuronal activity from several brain regions [31–33]. The LORETA-Key software is freely available (https://www.uzh.ch/keyinst/NewLORETA/Software/Software.htm).

EEG electrodes coordinates employed by the software are based on the MRI anatomical template from Montreal Neurological Institute (MNI152) which slice and classify the neocortical volume (limited to the gray matter) in 6239 voxels of dimension 5mm^3^ [34–36].

However, due to the volume conduction and the low spatial resolution of EEG, the dependence measures are highly contaminated with instantaneous, non-physiological effects. In this sense, was developed of capable coherence measures that appraise the lagged connectivity between various brain locations to verify the existence of distributed cortical networks. Lagged coherence connectivity expresses the coherence measured by the corrected standardized covariance of scalp electric potentials, extracting the instantaneous linear dependence [33, 37]. The lagged coherence becomes the adequate way to measure electrophysiological connectivity, removing the confounding effect of instantaneous dependence due to volume conduction and low spatial resolution [33].

To generate the regions of interest (ROIs) a voxel-wise approach was implemented, MNI coordinates of the areas under the electrode were determined by sLORETA. A size 10-mm-diameter sphere was defined from the seed points centered in the following ROIs [38, 39]: left and right primary somatosensory cortex (BA01) [40], the left and right insular cortex (BA47-BA48) [41, 42], the left and right anterior cingulate cortex (BA24) [43], and the left and right dorsolateral prefrontal cortex (BA09-BA10-BA46) [43], according to previous studies on the pain network. The coordinates are shown in Table 1.

**Table 1:** Montreal Neurological Institute (MNI) coordinates of regions of interest (ROI).

Seed	MNI coordinates			Brodmann Area
	x	y	z	
Left S1	-48	-24	52	BA01
Right S1	52	-16	44	
Left INS	-48	12	-2	BA47 and BA48
Right INS	36	6	6	
Left ACC	-2	8	30	BA24
Right ACC	1	8	30	
Left DLPFC	-2	46	-16	BA09, BA10 and BA46
Right DLPFC	2	46	-16	

ACC stands for anterior cingulate cortex, INS for insula, DLPFC for dorsolateral prefrontal cortex, and S1 for primary somatosensory.

The artifact-free EEG intervals were converted into ASCII files and then put into the sLORETA software. The segmented EEG intervals recorded in resting-state were analyzed by means of a fast Fourier transform algorithm, with 2 seconds of intervals according to the following frequencies: delta (2–3.5 Hz), theta (4–7.5 Hz), alpha-1 (8–10 Hz), alpha-2 (10.5-12Hz), beta-1 (13–18 Hz), beta-2 (18.5–21 Hz), beta-3 (21.5–30 Hz), gamma (30.5–44 Hz) [26]. The sLORETA functional images and color maps of lagged coherence were created in BrainNet Viewer, a graph-theoretical network visualization toolbox developed using MATLAB as a programming language in an easy, flexible, and quick manner. This software is freely available on the Neuroimaging Informatics Tools Resources Collabolatory website (NITRC): www.nitrc.org/projects/bnv/ [44].

#### 2.4.3 Pain measures and correlated symptoms, sleep quality, depressive symptoms, socio-demographic variables, clinical and psychiatric disorders, central sensitization, and serum BDNF

All instruments utilized to evaluate pain and correlated measures, psychological tests, and sleep quality are validated for the Brazilian population, including the Fibromyalgia Impact Questionnaire (FIQ) [45], Beck Depression Inventory-II [46], the Brazilian Pain Catastrophizing Scale (BP-PCS) [47], and the Pittsburgh Sleep Quality Index (PSQI) [48].

- The Fibromyalgia Impact Questionnaire (FIQ) is a self-administered instrument that includes 10 questions to assess the impact of FM on the quality of life. First question includes 11 items on a 4 point Likert type scale that evaluates how symptoms interfere with daily activities. Questions 2 and 3 ask about how patients felt daily and work disability, including housework. Questions 4 to 10 are horizontal linear scales that assess pain, fatigue, morning tiredness, stiffness, anxiety and depression. The maximum score is 100, the average scores around 50, and severity scores over 70 [45, 49].
- To evaluate global pain experienced over a 24-hour period throughout the four weeks of treatment, a Numeric Pain Scale (NPS) was utilized. The NPS measures pain intensity on a scale from 0 to 10, where 0 represents no pain and 10 represents the worst pain experienced in the majority of the 24-hour period across most days of the week [23, 24].
- The Pittsburgh Sleep Quality Index (PSQI) evaluates sleep quality and disturbances over the past month. The sum of the scores from seven components yields the global PSQI score, which ranges from 0 to 21 [48].
- The Beck Depression Inventory—Second Edition (BDI-II) is a self-report instrument consisting of 21 items. Its purpose is to measure the intensity of depression [46].
- The Brazilian Portuguese translation of the Pain Catastrophizing Scale (BP-PCS) comprises 13 items divided into three domains: magnification, helplessness, and rumination. The questions aim to assess the patient’s feelings and thoughts related to pain and measure pain-related catastrophizing [47].
- Enzyme-linked immunosorbent assay (ELISA) was used to measure blood levels of BDNF. Monoclonal antibodies specific for BDNF were employed from R&D Systems (MN, USA; ChemiKine BDNF Sandwich ELISA kit, CYT306; Chemicon/Millipore, Billerica, MA, USA). The inter-assay variance was evaluated using two plates per kit on two different days within the same week. All procedures followed the manufacturer’s recommendations, and the lowest detection limit for BDNF was 7.8 pg/ml. Optical density measurements at 450 nm were conducted using the Promega GloMax®-Multi Microplate Reader. Additionally, the Bio-Plex®-200 instrument from Bio-Rad was used for multiplexing assay measurements. Total protein was assessed using bovine serum albumin following the Bradford method. A standardized questionnaire was utilized to collect demographic data and medical comorbidities. Patients were asked to furnish details concerning their age, gender, educational background, marital status, and lifestyle choices. Additionally, they provided information about their overall health condition, including clinical and psychiatric diagnoses.
- Mini-International Neuropsychiatric Interview (MINI) is a short (15–30 min) structured psychiatric diagnostic interview aimed to screen for DSM-IV and ICD-10 diagnoses [50].
- The central sensitization inventory (CSI) is a tool that identifies key symptoms related to central sensitization processes by quantifying the severity of these symptoms. It consists of two parts: Part A is a 25-item self-report questionnaire designed to assess symptoms related to health and Part B (not rated) is designed to determine if one or more specific disorders [51].

### 2.5 Sample size justification

The initial sample size was determined based on a previous study [52], which investigated the impact of a single session of a-tDCS on the DLPFC using EEG resting-state measurements in healthy subjects. This study reported an effect size (η^2^ = 0.10) in the theta frequency band for the active tDCS group compared to the sham group. To estimate our sample size, we planned an analysis of variance (ANOVA) with a one-way repeated measures design, considering both within-group and between-group effects. The effect size for the ANOVA was set at f = 0.33, corresponding to the previously reported effect size (η^2^ = 0.10). With a significance level (α) of 0.05 and a power (1-β) of 0.90, we determined a sample size of 40 patients, the noncentrality parameter (γ) equal to 17.42 and the critical F value equivalent to 2.86. We added 20% to account for potential dropouts, resulting in a final sample size of 48 patients. These patients were divided into four groups, with 16 patients in each HB-a-tDCS group targeting the M1 and DLPFC regions and eight patients in each of the HB-s-tDCS groups targeting the same regions. To ensure our study had adequate statistical power to detect significant effects of the intervention, we assessed the power of these analyses focusing on primary outcomes, particularly differences in lagged coherence connectivity before and after intervention. We observed significant differences between the active HB-a-tDCS and HB-s-tDCS groups targeting the DLPFC region. For instance, the mean difference in lagged coherence connectivity between the left ACC and right INS was -17.76 (SD = 20.05) for the HB-a-tDCS group, compared to 14.35 (SD = 17.79) for the sham group, resulting in a calculated effect size (d) of 1.69.

Similarly, comparing the mean difference in lagged coherence connectivity between the left DLPFC and left ACC, the HB-a-tDCS group (-40.59, SD = 111.0) showed a significantly different effect compared to the HB-s-tDCS group applied over M1 (-187.7, SD = 153.4), with an effect size (d) of 1.72. With a type I error rate of 0.05, the power for these outcomes was 97% and 92%, respectively. Estimation and power analysis were performed using G*Power 3 software [53, 54].

### 2.6 Randomization

Randomization was conducted in six blocks of eight patients to ensure unpredictability and maintain blinding during the allocation process. The randomization process involved utilizing an online tool (www.sealedenvelope.com) and establishing a 2:1:2:1 allocation ratio to explore HB-tDCS treatment dimensions and monitoring adverse effects [55], allocating participants into four groups: (1) HB-a-tDCS-DLPFC, (2) HB-s-tDCS-DLPFC, (3) HB-a-tDCS-M1, and (4) HB-s-tDCS-M1. An external, professionally conducted randomization and randomized number codes were sealed in envelopes with the patient’s entry sequence number. Two engineers, independent of patient assessments, were responsible for programming the intervention device. They opened the sealed envelope only after obtaining informed consent, ensuring that the allocation remained concealed until the appropriate time for the researchers involved in the patient’s evaluation.

### 2.7 Blinding

All participants were unaware of which stimulation type (active or sham) they received during the entire study sequence. The HB-s-tDCS group’s device was set up to provide the stimulation three times: in the initial 30 seconds, in the middle 30 seconds, and in the last 30 seconds of the stimulation. After the post-assessment, we evaluate the blinding effectiveness by asking all participants which stimulation they suppose they received, active or sham, and measuring their confidence level using a 5-point Likert scale.

### 2.8 Statistical analysis

Descriptive statistics were employed to summarize the main demographic characteristics of the sample. The Shapiro-Wilk test was used to assess the normality of data distribution of continuous variables. To compare continuous variables between groups, independent sample t-tests were utilized. The chi-squared and Fisher’s exact tests were employed for comparing categorical variables between groups. Within-group analyses were conducted using paired t-tests to examine differences between baseline and treatment end measures. All statistical analyses were performed using two-tailed tests at the 5% significance level with SPSS, version 22.0 (SPSS, Chicago, IL).

We used statistical nonparametric mapping (SnPM) with 5000 permutations using a t-statistic, with corrections for multiple comparisons to identify cortical voxels exhibiting significant differences in lagged coherence across the eight regions of interest (ROIs) within each frequency band and within and between groups. All analyses adhered to a 0.05 alpha’s error level. This nonparametric approach, rooted in permutation test theory, alleviates the Gaussian assumption requirement for correcting multiple comparisons [56].

For the secondary outcomes, the regression analyses were performed for the EO and EC conditions. The treatment effect within-groups considering the delta-value of lagged coherence connectivity for each frequency band between ROIs as the dependent variable and the delta-value for FIQ, BDI, BP-PCS, PSQI, and serum BDNF levels as independent variables. We build the correlation matrices for generating connectivity maps using the sLORETA package. A test with 5000 permutations was used to determine the significance threshold and correct for multiple comparisons. The regression analyses were conducted using two-tailed tests, and a type I error of 5% was accepted.

## 3. Results

### 3.1 Demographic and clinical characteristics of the subjects

We included 48 women with fibromyalgia. However, we examined a total of 43 participants who were randomly assigned to one of the following treatment groups: HB-a-tDCS on DLPFC (n = 16), HB-a-tDCS on M1 (n = 13), HB-s-tDCS on DLPFC (n = 8), or HB-s-tDCS on M1 (n = 6). Five patients were excluded from the analyzes. Table 2 provides an overview of the subjects’ baseline demographic and clinical characteristics.

**Table 2:** Demographic and clinical characteristics of the study sample are presented. Values are reported as mean (SD) for continuous variables, median (IQ) or % for categorical variables (n = 43).

	HB-a-tDCS DLPFC GROUP (N = 16)	HB-s-tDCS DLPFC GROUP (N = 8)	HB-a-tDCS M1 GROUP (N = 13)	HB-s-tDCS M1 GROUP (N = 6)
	Mean (SD) Median (IQ _25–75_)	Mean (SD) Median (IQ _25–75_)	Mean (SD) Median (IQ _25–75_)	Mean (SD) Median (IQ _25–75_)
**Demographic measures**				
Age (years)	48.75 (9.04) 47.50 (42.75, 56.50)	45.88 (10.41) 43.00 (36.25, 57.25)	49.38 (11.54) 53.00 (38.00, 59.00)	44.17 (9.06) 42.50 (35.50, 55.00)
Years of formal study	12.00 (3.83) 11.00 (9.25, 16.00)	12.50 12.00 (9.50, 16.00)	11.62 (3.79) 11.00 (10.00, 14.50)	13.83 (3.31) 14.50 (11.75, 16.25)
NPS (Numerical Pain Scale)	8.47 (1.30) 8.60 (7.47, 9.60)	8.66 (1.02) 9.11 (7.44, 9.42)	8.19 (1.52) 8.10 (7.10, 9.65)	7.13 (1.48) 7.85 (6.10, 8.02)
American College of Rheumatology (ACR) diagnosis tool	23.38 (3.79) 23.00 (21.00, 25.00)	24.50 (3.38) 24.00 (21.75, 26.00)	23.50 (2.71) 24.00 (21.50, 25.75)	20.83 (3.48) 20.00 (18.50, 23.50)
Employed ^a^	11/5 (68.8%)	5/3 (62.5%)	7/6 (53.8%)	3/5 (50.0%)
Smoking ^a^	4/12 (25%)	1/7 (12.5%)	3/10 (23.1%)	1/5 (16.7%)
Drinking ^a^	7/9 (43.8%)	4/4 (50.0%)	6/7 (46.2%)	2/4 (33.3%)
**Clinical comorbidity**				
Hypertension ^a^	6/10 (37.5%)	2/6 (25.0%)	3/10 (23.1%)	2/4 (33.3%)
Cardiac Disease ^a^	0/16 (0.0%)	0/8 (0.0%)	1/12 (7.7%)	1/5 (16.7%)
Diabetes Disease ^a^	2/14 (12.5%)	1/7 (12.5%)	0/13 (0.0%)	1/5 (16.7%)
Hypothyroidism ^a^	3/13 (18.8%)	2/6 (25.0%)	2/11 (15.4%)	1/5 (16.7%)
Asma ^a^	6/10 (37.5%)	1/7 (12.5%)	6/10 (37.5%)	1/5 (16.7%)
Epilepsy ^a^	0/16 (0.0%)	0/8 (0.0%)	0/13 (0.0%)	0/6 (0.0%)
Renal Insufficiency ^a^	0/16 (0.0%)	1/7 (12.5%)	0/13 (0.0%)	0/6 (0.0%)
**Biochemical measures**				
Serum BDNF (Brain-derived neurotrophic factor) (pg/ml)	51.66 (42.49) 31.48 (19.12, 89.20)	31.67 (25.76) 26.49 (11.54, 42.13)	39.17 (18.46) 30.67 (24.28, 58.36)	40.98 (36.94) 23.78 (14.75, 86.57)
**Mood, pain-related and sleep quality measures**				
Beck Depression Inventory (BDI)	24.56 (13.25) 21.50 (13.50, 37.00)	28.25 (8.56) 28 50 (21.50, 36.50)	22.46 (9.49) 22.00 (14.50, 29.00)	16.33 (8.91) 14.00 (10.50, 23.00)
Brazilian Portuguese Central Sensitization Inventory (BP-CSI)	65.00 (15.90) 63.50 (52.25, 79.75)	64.75 (8.27) 65.50 (58.25, 73.00)	62.92 (15.46) 69.00 (48.00, 77.00)	56.67 (9.11) 59.00 (49.25, 63.25)
Brazilian Portuguese Pain Catastrophizing Scale (BP-PCS)	34.25 (13.09) 35.50 (29.75, 42.00)	38.63 (9.72) 41.00 (33.50, 43.50)	38.23 (7.99) 41.00 (32.00, 45.00)	27.17 (8.65) 27.50 (18.75, 32.25)
Pittsburgh Sleep Quality Index (PSQI) (total score)	12.44 (4.09) 13.50 (9.50, 15.75)	11.75 (4.49) 13.50 (6.50, 14.75)	12.54 (3.25) 13.00 (9.50, 13.50)	10.67 (1.36) 10.50 (9.75, 11.50)
Fibromyalgia Impact Questionnaire (FIQ) (total score)	68.58 (17.00) 76.06 (62.05, 79.59)	69.78 (9.83) 72.30 (67.98, 76.31)	68.94 (16.58) 68.68 (59.18, 81.30)	55.59 (19.82) 61.51 (37.21, 71.87)
**Psychiatric disorder**				
Major Depressive Disorder (MDD)(current) ^a^^,^^b^	10/6 (62.5%)	5/3 (62.5%)	6/6 (50.0%)	1/5 (16.7%)
Generalized Anxiety Disorder (GAD) ^a,c^	4/11 (26.7%)	2/6 (25.0%)	1/11 (8.3%)	3/3 (50.0%)
**Medication**				
Antidepressant ^a^	11/5 (68.8%)	5/3 (62.5%)	7/6 (53.8%)	5/1 (83.3%)
Anticonvulsivant ^a^	3/13 (18.8%)	3/5 (37.5%)	2/11 (15.4%)	3/3 (50.0%)
Benzodiazepines ^a^	4/12 (25%)	2/6 (25.0%)	0/13 (0.0%)	1/5 (16.7%)
Opioid analgesic ^a^	6/10 (37.5%)	2/6 (25.0%)	2/11 (15.4%)	2/4 (33.3%)
Non-opioid analgesic ^a^	16/0 (100%)	8/0 (100%)	12/1 (92.3%)	6/0 (100%)
Antihypertensive ^a^^,^^b^	6/9 (40.0%)	2/6 (25.0%)	4/9 (30.8%)	3/3 (50.0%)

a–Values reported as relative frequency (Yes/No) and percentage (%) for categorical variable performed using Chi-square test.

^b^ n = 42.

^c^ n = 41

### 3.2 Primary outcome

#### 3.2.1 Effect of treatment between-groups on lagged coherence connectivity in EC and EO conditions

**Fig 2:**
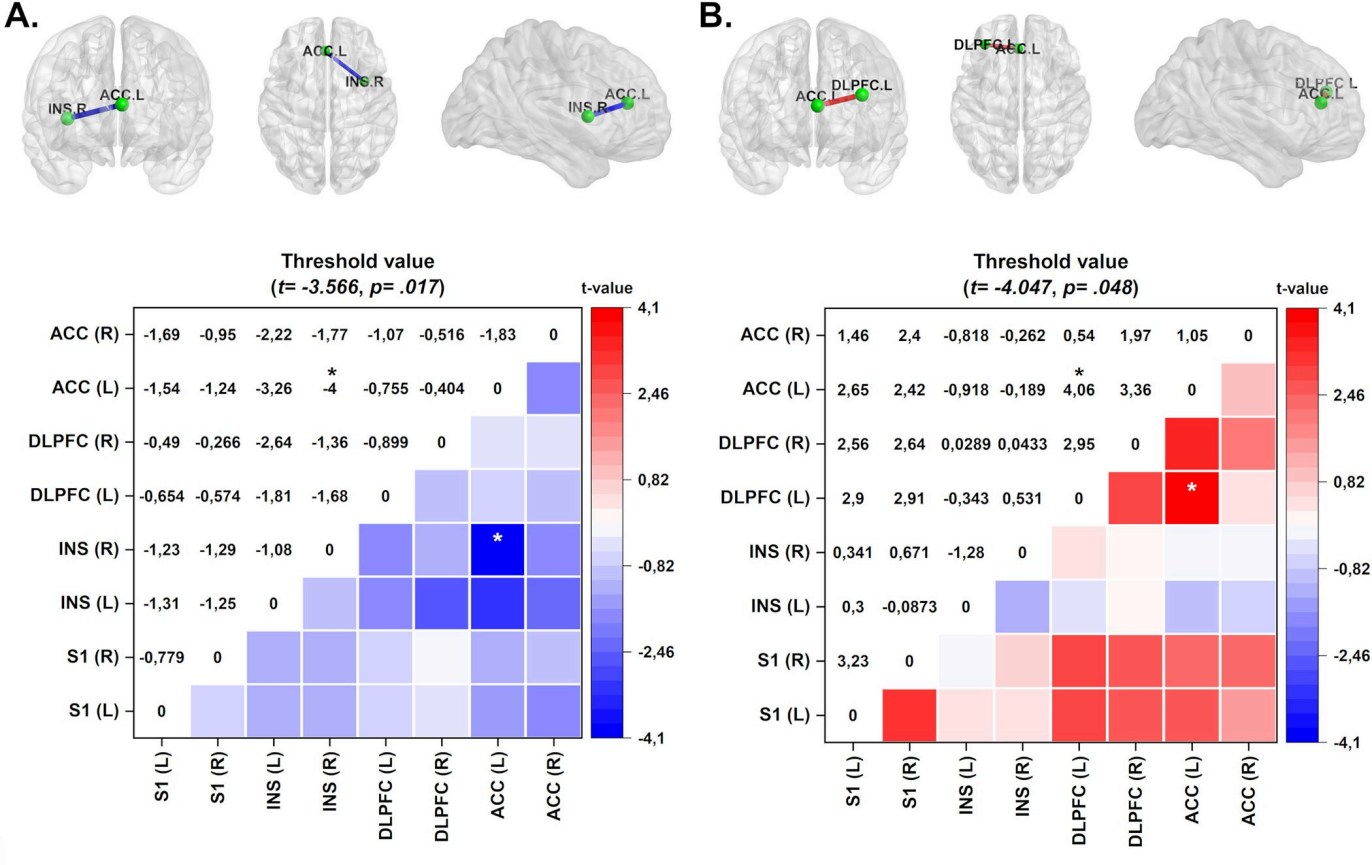
Connectivity (frontal, axial, and sagittal planes) and color maps of between-group analysis of the tDCS intervention. The colored edge represents connections with significant differences. (A) Maps shows decreased connectivity between left ACC and right INS in the HB-a-tDCS on DLPFC group in the delta frequency band which present large effect size (Cohen’s d = 1.69). (B) HB-a-tDCS over DLPFC group exhibits increased connectivity in the theta frequency band between the left DLPFC and left ACC, with large effect size (Cohen’s d = 1.72). Effect sizes are based on Cohen’s d: small = 0.2, medium = 0.5, large = 0.8. Values are given in t-values. ACC = anterior cingulate cortex; INS = insula; DLPFC = dorsolateral prefrontal cortex; SI = primary somatosensory cortex. *p<0.05.

In the EC condition, when it was assessed the comparisons of lagged coherence connectivity expressed by Δ-means (pre-minus post-treatment) according to the treatment group (HB-a-tDCS and HB-s-tDCS) applied over left DLPFC, we found that the HB-a-tDCS over left DLPFC decreased connectivity between right insula and left ACC in the delta frequency band (see Fig 2A).

In the EO condition, when it was assessed, the comparison of lagged coherence connectivity expressed by Δ-means (pre-minus post-treatment) according to the treatment group HB-a-tDCS applied over the left DLPFC exhibits increased connectivity in the theta frequency band between the left DLPFC and left ACC compared with HB-s-tDCS (pre-minus post-treatment) over M1 (see Fig 2B). In contrast, in the EC condition, we did not find a difference between these groups.

The lagged coherence analysis in EO and EC conditions doesn’t show differences in the HB-a-tDCS pre- to post-treatment (Δs) between DLPFC compared to M1, as well as between HB-a-tDCS over M1 compared with HB-s-tDCS over DLPFC.

#### 3.2.2 Effect of treatment within-groups on lagged coherence connectivity in EC and EO conditions

**Fig 3:**
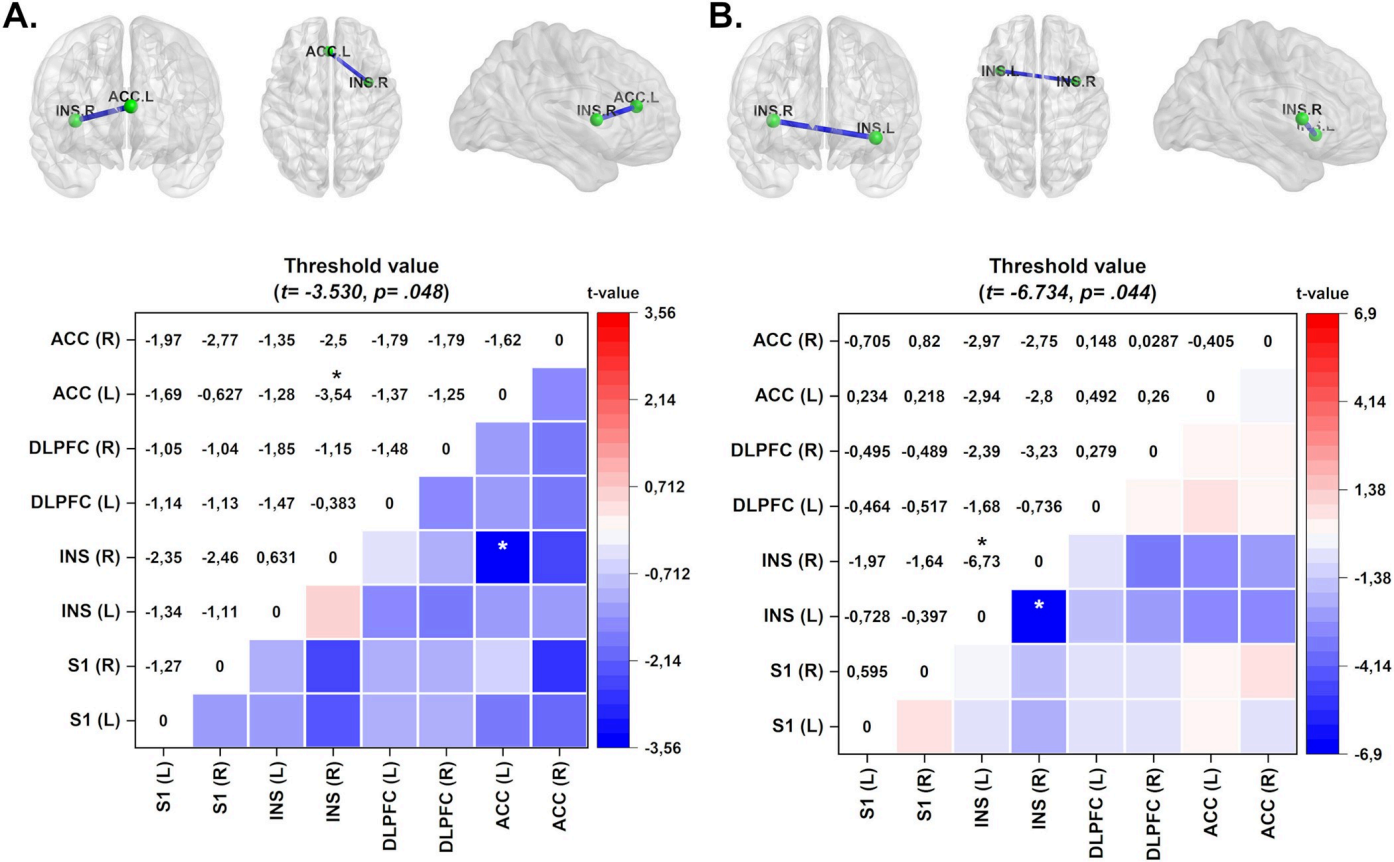
Connectivity maps and color maps of within-group analysis of the tDCS intervention. The colored edge represents connections with significant differences. Data represent the delta-value of the mean oscillation of the frequency band from pre- to post-treatment in each brain region. (A) The maps demonstrate decreased connectivity between the left ACC and right INS in the HB-a-tDCS on DLPFC group in the delta frequency band in EO condition which present moderated effect size (Cohen’s d = 0.70). (B) The HB-s-tDCS on M1 group exhibits decreased connectivity in the gamma frequency band between the right and left INS with large effect size (Cohen’s d = 2.55). Effect sizes are based on Cohen’s d: small = 0.2, medium = 0.5, large = 0.8. Values are given in t-values. ACC = anterior cingulate cortex; INS = insula; DLPFC = dorsolateral prefrontal cortex; SI = primary somatosensory cortex. *p<0.05.

In the EC condition, when it was assessed the comparisons of lagged coherence connectivity expressed by Δ-means (pre-minus post-treatment) according to the treatment group (HB-a-tDCS and HB-s-tDCS) applied over target brain areas nominally left DLPFC and M1, we found that the HB-a-tDCS over left DLPFC decreased connectivity between right insula and left ACC in the delta frequency band (see Fig 3A). In EC and EO conditions, statistical significance was not identified in the HB-s-tDCS over DLPFC, and in HB-a-tDCS over M1, for all frequency bands.

In the EO condition, when it was assessed the comparisons of lagged coherence connectivity expressed by Δ-means (pre-minus post-treatment) HB-s-tDCS over on M1 demonstrated decreased lagged coherence connectivity between left and right insula in the gamma frequency band in EO condition (see Fig 3B).

### 3.3 Secondary outcomes–exploratory analysis

#### 3.3.1 Effect of treatment within-groups on the delta-value lagged coherence connectivity in the EC and EO conditions, sleep quality and pain catastrophizing

We conducted a series of linear regression analyses to investigate the relationship between ROI connectivity in specific frequency bands (delta, theta alpha, beta, and gamma) and various factors such as depressive symptoms, pain-related measures, sleep quality, and serum BDNF levels. The PSQI and BP-PCS were the variables that showed a statistically significant correlation with the frequency bands, considering either EO or EC conditions. When comparing the delta values (from before to after) between the HB-a-tDCS and HB-s-tDCS over DLPFC with PSQI, a significant effect was found, with Cohen’s *d* values are *d* = 0.68 and *d* = 0.62, respectively. In contrast, we did not find a significant effect either on HB-a-tDCS or HB-s-tDCS applied on M1.

The delta value (from before to after) in the BP-PCS was significant both HB-a-tDCS and HB-s-tDCS over DLPFC, with large effect sizes of *d* = 1.19 and *d* = 1.35, respectively. The effect sizes for HB-a-tDCS and HB-s-tDCS on M1 were moderate, with *d* = 0.40 and *d* = 0.51, respectively. The effect size to remaining variables are available in the supporting data (S1 Table).

**Fig 4:**
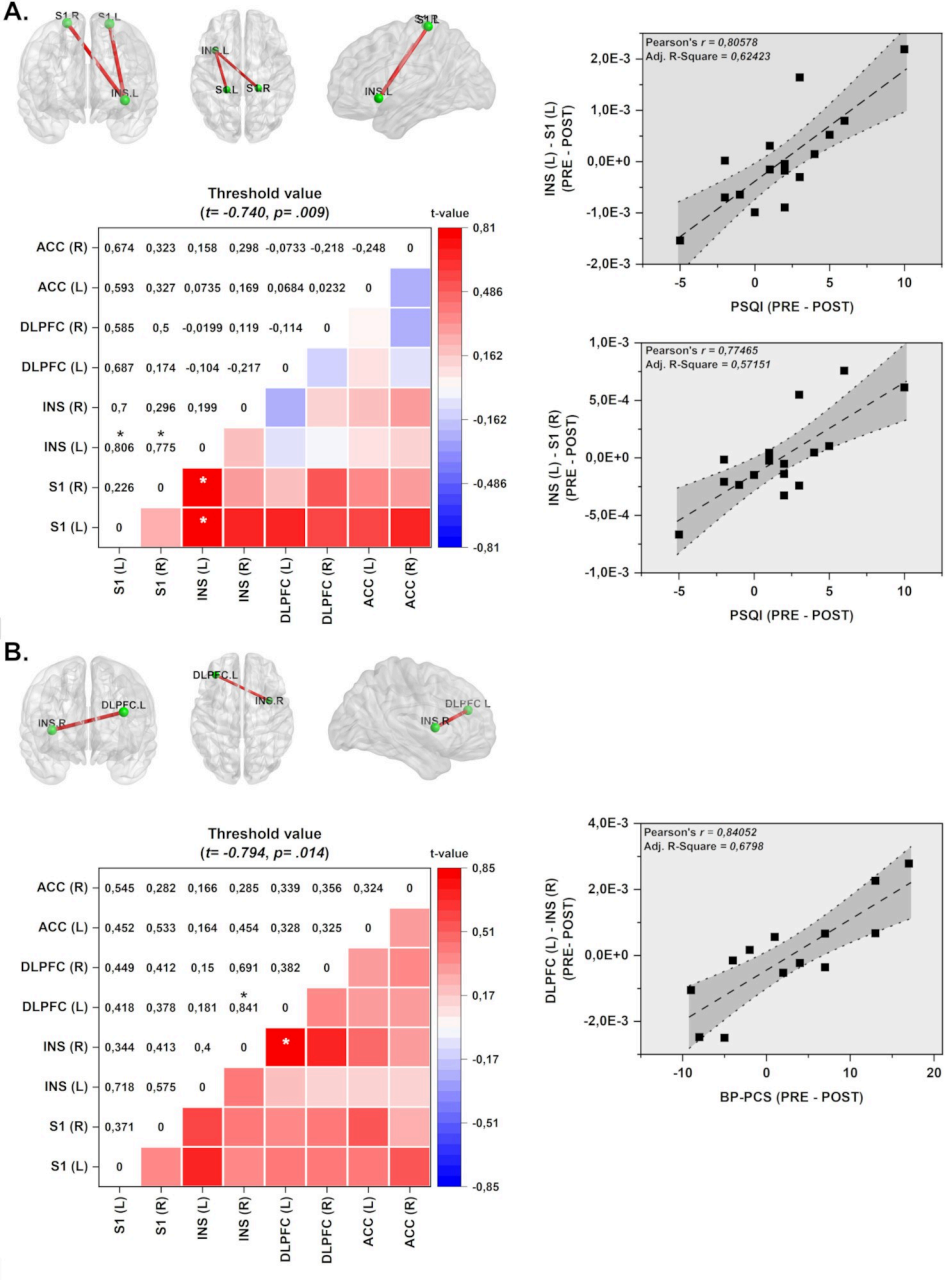
Connectivity and color maps of the linear regression analyses considering Δ-values of differences pre- to post-treatment of ROI connectivity. The Δ-values of ROI connectivity were correlated with Δ-values of the following variables: depressive symptoms, pain-related, sleep quality, and serum BDNF. (A) The HB-a-tDCS over DLPFC group presented increased lagged coherence connectivity in the beta-3 band between left INS and left and right S1, which is correlated with sleep quality measures (PSQI) in EO condition. (B) The HB-a-tDCS over M1 group shows increased coherence connections in the delta frequency band between right INS and left DLPFC, which is correlated with pain catastrophizing scores (BP-PCS) in the EC condition. Values given in Cohen r = correlation coefficient (small = 0.1; medium = 0.3; large = 0.5). ACC = anterior cingulate cortex; INS = insula; mPFC = medial prefrontal cortex; S1 = primary somatosensory cortex. *p<0.05.

In the EO condition, where participants received HB-a-tDCS over the DLPFC, we observed a positive correlation between sleep quality and the lagged coherence connectivity in the beta-3 frequency band. Specifically, this correlation was observed between the left insula and the left and right primary somatosensory cortex (S1) (see Fig 4A).

In the EC condition, where participants received HB-a-tDCS over the M1, we found that change of pain catastrophizing form pre to treatment end was positively correlated with the lagged coherence connectivity in the delta frequency band. This correlation was observed between the right insula and the left DLPFC (see Fig 4B).

### 3.4 Adherence, compliance, and side effects across the treatment

**Fig 5:**
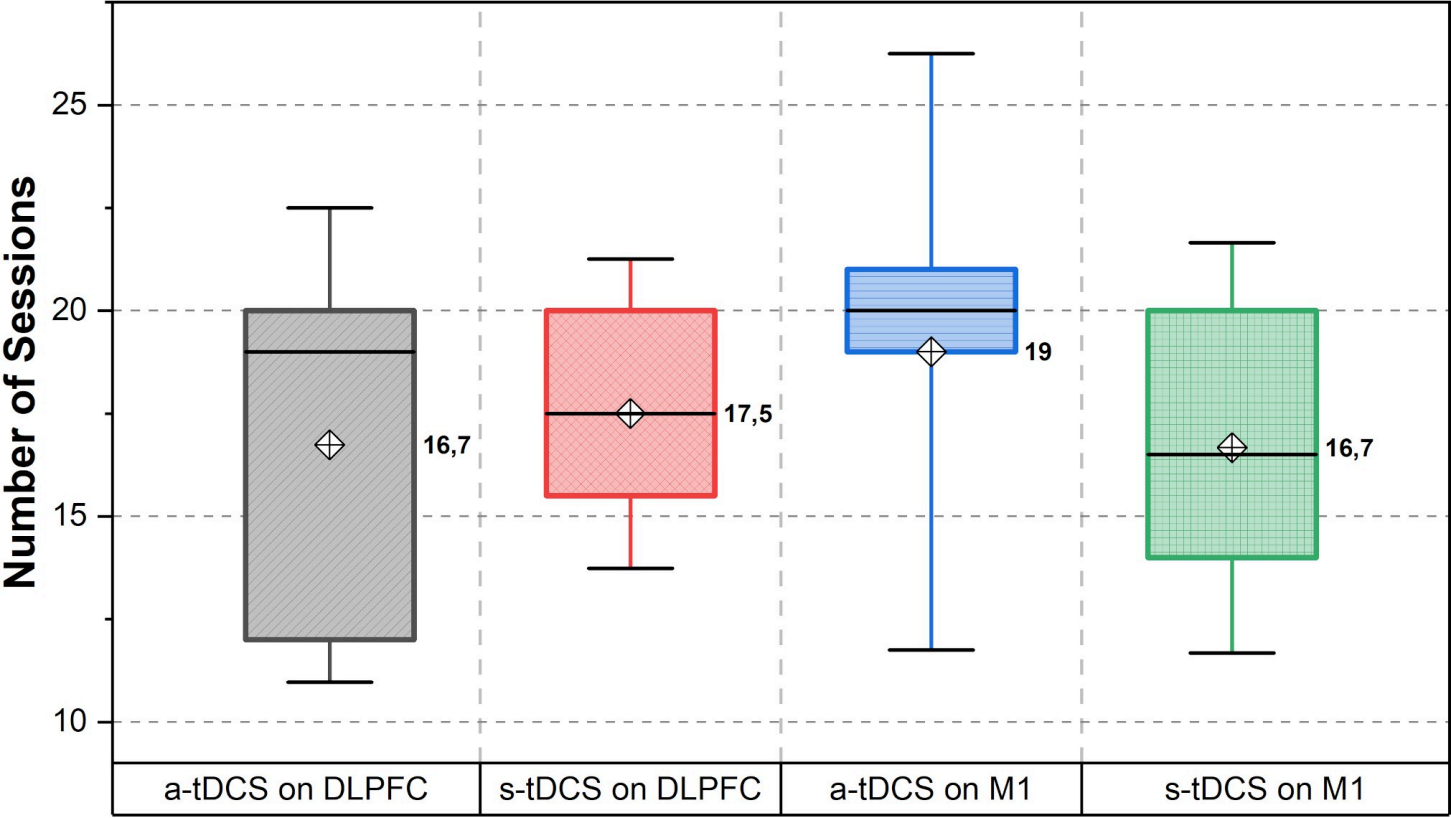
Number of sessions according to HB-a-tDCS on DLPFC, HB-s-tDCS on DLPFC, HB-a-tDCS on M1, and HB-s-tDCS on M1.

The data in Fig 5 provides information on the average number of valid sessions completed per group, indicating the level of adherence and compliance. The mean (SD) values reflect the average number of valid sessions completed, while the median (IQ_25-75_) values give the middle range of valid session counts within each group. According to the total sample size, we planned 860 HB-tDCS sessions and recorded 758 valid sessions, corresponding to 88.1% of the total amount.

We predicted 320 sessions for the HB-a-tDCS over the DLPFC group (n = 16) and conducted 271 valid sessions that comprised 84.68% of the total. In the group where HB-s-tDCS was applied over DLPFC (n = 8), 140 of the 160 estimated sessions were completed, equivalent to 87.5%. For the group stimulated with HB-a-tDCS on M1 (n = 13), we administered 247 sessions of the 260 determined, corresponding to 95% of the total sum. Finally, of the 120 sessions stipulated by the HB-s-tDCS on the M1 group (n = 6), 100 were performed, computing 83.33%.

The percentage of patients who received HB-a-tDCS and correctly guessed the stimulation condition was 89% for the M1 anode position and 91.1% for the left DLPFC anode position. In contrast, those who received HB-s-tDCS on M1 correctly guessed 18.5%, and on left DLPFC the percentage was 14.7%. Regardless of the stimulation site, participants reported a confidence level of 89.6% for HB-a-tDCS and 84.5% for HB-s-tDCS.

**Fig 6:**
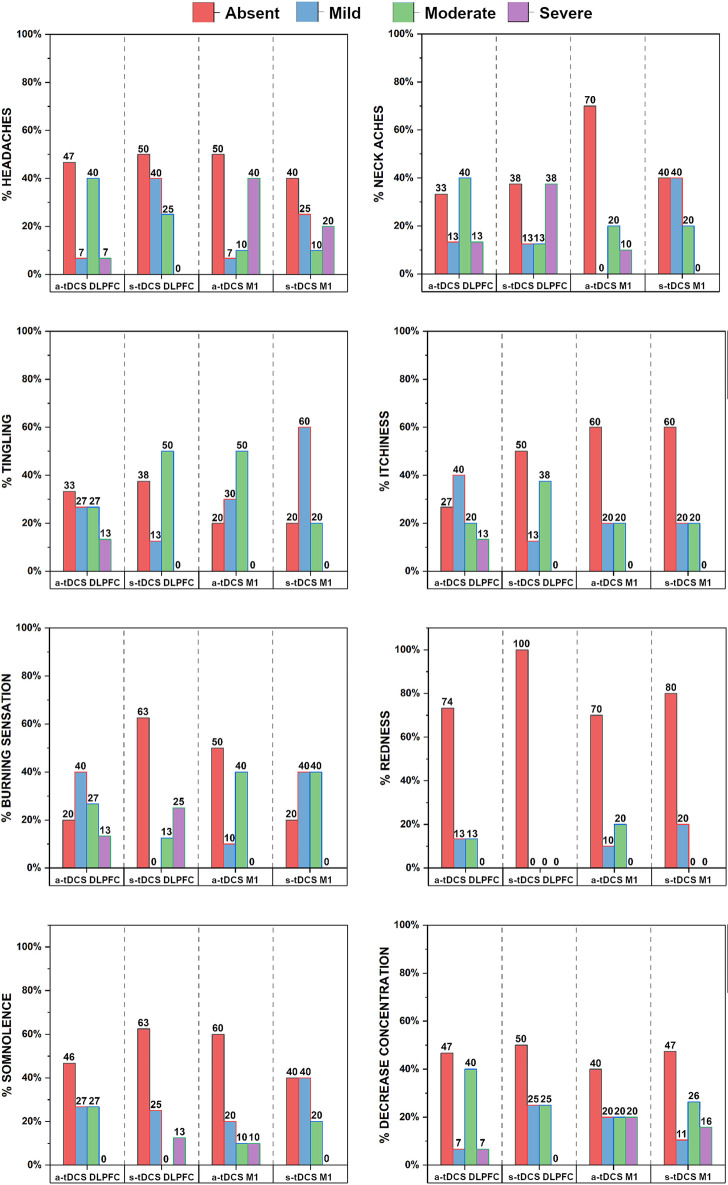
Side effects are presented as percentages (%).

The side effects questionnaire included inquiries about various symptoms, including headache, neck aches, tingling, redness, burning sensation, itching, somnolence, and decreased concentration. The Fig 6 illustrates the reported symptoms and their severity classified as absence, mild moderate and severe. It is important to note that no participants discontinued the treatment due to uncomfortable side effects.

## 4. Discussion

The unique aspect of this multi-group trial is its focus on the effects of HB-tDCS on specific frequency bands of EEG within brain areas involved in pain processing. In the EC condition, HB-a-tDCS over left DLPFC reduced lagged coherence connectivity in the delta frequency band between the right insula and left ACC compared to HB-s-tDCS. In the EO condition, HB-a-tDCS over left DLPFC increased lagged coherence connectivity between the left DLPFC and left ACC in the theta band compared to HB-s-tDCS applied over M1. The study revealed that HB-a-tDCS over the DLPFC group exhibited enhanced connectivity in the beta-3 band between the left insula and bilateral S1, correlating with sleep quality measures, while HB-a-tDCS over the M1 group showed increased coherence connections in the delta frequency band between the right insula and left DLPFC, associated with pain catastrophizing scores.

### 4.1 Brain connectivity and neurophysiology

#### 4.1.1 HB-tDCS effects in brain connectivity between and within-groups

It was found that HB-a-tDCS over the left DLPFC reduced the connectivity between the left ACC and the right insula in the delta frequency band in EC condition. These brain regions play crucial roles in cognitive and emotional processing, particularly in pain perception, where the insula integrates sensory, affective, and cognitive aspects [57]. Increased glutamate levels in the insula contribute to heightened pain sensitivity [58]. The novelty of these findings helps us understand how HB-a-tDCS changes the brain’s pain processing. The interaction between the insula and ACC, both involved in sensory and affective aspects of pain, is vital for modulating the subjective pain experience [59]. This study suggests that HB-a-tDCS may change the neuroplasticity processes that control pain processing and other related symptoms. This is because the insula is connected to many parts of the brain, including the DLPFC.

In the EO condition, the HB-a-tDCS applied over the left DLPFC exhibited increased connectivity in the theta frequency band between the left DLPFC and left ACC. This finding suggests that HB-a-tDCS may operate directly over the stimulation site and in the brain networks related to her and how information processing is integrated between these brain areas linked to controlling emotions and cognition [60]. The increased coupling between these regions indicates that treatment effects might improve the maladaptive regulation of cognition and emotions. These findings align with a meta-analysis that supports the idea that higher activity in the ACC is associated with a better response to antidepressants. This includes selective serotonin reuptake inhibitors (SSRIs), atypical antidepressants, and ketamine [60]. So, the current study sheds light on the effects of HB-a-tDCS on brain regions connected to dysfunctional neurobiological processes, particularly on the left side, which are prevalent in chronic pain and fibromyalgia [59]. Previous research in FM has shown that decreased activity in these areas is associated with difficulties in attention control, heightened pain perception, and emotional disturbances [61, 62]. While these connectivity measurements provide valuable insights, they are inferential and support the notion that a-tDCS’s effect on the left prefrontal cortex (PFC) neural network could ameliorate the maladaptive neuroplasticity underlying FM symptoms. The fact that the left DLPFC and left ACC were more connected in the theta frequency band suggests that these areas can talk to each other better, which is probably essential for processing emotions and improving cognitive function [63]. This finding is consistent with the theory that modulating ACC activity can impact the emotional aspects of pain perception [63]. However, it is crucial to exercise caution in interpreting these results, as further larger studies is necessary to ascertain whether the remapping of neural networks in pain processing pathways significantly impacts patient-reported outcome measures (PROM).

We found that before to after HB-a-tDCS over DLPFC in EC condition, there was a decrease in the connectivity between the left ACC and right insula in the delta frequency band. This result indicates that a-tDCS may change how affective and attentional circuits work. These results align with earlier research that showed controlling excitatory activity in ACC neurons was needed to improve negative emotions in people with chronic pain [64, 65]. They also provide evidence supporting the biological link between HB-a-tDCS effects and FM symptoms, including improvements in cognitive impairment, pain exaggeration, and depressive symptoms [13, 14, 18]. Moreover, earlier studies have linked oscillations in the delta frequency with cognitive functions such as attention, working memory, and emotional regulation, further supporting this conclusion [66].

In the HB-s-tDCS on M1 under EO conditions, we observed a decreased connectivity in the gamma frequency band between the right and left insula. This reduction suggests a decrease in synchronized brain activity. Gamma waves, characterized by rapid brain activity, are influenced by various factors, including the balance of excitatory and inhibitory neurotransmission, particularly involving gamma-aminobutyric acid (GABA). These oscillations are associated with cognitive processes such as attention, working memory, sensory perception, and memory formation and retrieval. According to recent meta-analyses, chronic pain may disrupt the production of gamma-band oscillations, making it difficult for individuals to relate to pain perception [67]. Additionally, this result may be influenced by a potential placebo effect. Previous literature suggests that the placebo effect observed in randomized controlled trials may reflect the influence of individuals believing they are receiving an active treatment [68]. Positive expectations and beliefs associated with receiving an active treatment can lead to neurotransmitter release, affecting neural activity and cognitive processing. Due to the limitations of measures used to evaluate the neural system involved in the excitability state, further studies using complementary measures, such as EEG and functional neuroimaging, are needed to understand the neurobiological mechanisms underlying the observed findings and their implications for pain perception.

#### 4.1.2 HB-tDCS effects in brain connectivity correlation with neurophysiological measures

Our study found that applying HB-a-tDCS to the DLPFC significantly decreased PSQI scores, an effect related to the amplitudes of the beta-3 band under EO conditions. Interestingly, we observed a positive correlation between connectivity in the left insula and the S1 and improved sleep quality scores. The literature about the connectivity of S1 and chronic pain symptoms has found cumulative data pointing out that S1 is likely a key area associated with changes in brain connectivity and pain-related clinical symptoms. Aligning with this perspective, previous research in FM revealed increased connectivity between S1 and the bilateral anterior insula during the pain phase [69, 70]. Similarly, a chronic low back pain (cLBP) study showed heightened connectivity between the S1 and anterior insula cortex. Another study in FM using fMRI found that reduced connectivity between the left S1 and the PAG is associated with the inefficiency of the descending pain inhibitory system (DPIS) [11]. This reduced connectivity was related to worse sleep quality and pain severity [11]. Although the precise mechanisms underlying active tDCS to improve sleep quality remain unclear, changes in connectivity within somatosensory cortex-related networks suggest a potential role for HB-a-tDCS in improving dysfunctional neural processing involving this area.

The HB-a-tDCS on M1 influenced the lagged coherence connectivity in the delta frequency, leading to a decrease in pain catastrophizing. This significant result suggests that patients with enhanced connectivity between these brain regions may experience a reduction in pain catastrophizing [71]. These findings, which align with previous research on the relationship between pain catastrophizing and pain perception, particularly in areas such as the insula [72–74], underscore the importance of understanding the interplay between pain catastrophizing and the neural mechanisms in pain perception. Similar outcomes were observed in delta oscillation, which is linked to attentional processes and the modulation of internal and external stimuli associated with fundamental biological motivations [75]. However, further research is needed to fully understand the mechanisms behind these findings and to determine whether brain oscillation bands could serve as treatment response indicators in chronic pain patients.

Our study found that applying HB-a-tDCS over M1 led to significant improvements in FIQ, NPS, and BDI scores, aligning with earlier studies that have identified M1 as a critical target for HB-a-tDCS in reducing pain and associated symptoms in FM [17, 18, 76]. Similarly, applying HB-a-tDCS to the l-DLPFC led to lower levels of NPS and BDNF in the blood, which supports the DLPFC as a target for pain reduction stimulation in FM [76]. Our findings also suggest that BDNF levels might indicate HB-a-tDCS effects on pain scores. However, it’s important to note that there was a significant reduction in serum BDNF during treatment, regardless of the treatment group. It seems that the effects of tDCS alone may not completely account for the decrease in BDNF levels. Other factors, like patient-clinician interactions, treatment expectations, motivation, and genetic variations, may also play a role in the neuroplasticity processes [77].

The group that received HB-s-tDCS on the DLPFC showed improvements in pain scores and depression symptoms (refer to S1 Table). We don’t have a clear explanation for these results, the literature suggests that the placebo effect in randomized controlled trials can stem from individuals believing they are receiving active treatment [68]. Other factors, such as disease progression, symptom fluctuations, regression to the mean, and response bias in self-reported symptoms, can also magnify the placebo effect [68]. A meta-analysis found that the placebo effect is usually tiny and connected to subjective outcomes [78]. However, studies with depressed patients who did not respond to treatment showed a significant placebo effect with a large effect size across many therapies, including tDCS [79]. This evidence suggests that the placebo effect is natural and may vary depending on the treatment type. Our findings highlight the need for more research to understand the interplay of factors contributing to the placebo effect and its implications for developing effective clinical treatment plans.

### 4.2 Clinical relevance

We employed a pre-planned mITT analysis in this study with an adjusted protocol, encompassing all patients who completed at least 10 sessions. We use the most of the literature supporting clinical symptom improvement in chronic pain as a guide when choosing the ten ITT sessions [76]. Although this method is not as strict as classical ITT, it suggests that HB-a-tDCS could be useful in clinical settings with a minimum number of sessions. The improvement in clinical measures aligns with changes in neurophysiological measures, which can serve as neural markers of the processes underlying chronic pain. So, they support linking the neurophysiological effects with the clinical effects of HB-tDCS in the treatment of FM. They also demonstrated that HB-tDCS is safe, suggesting it is a viable treatment option for FM by altering neural networks associated with chronic pain in FM and its symptoms.

### 4.3 Study limitations

The main concerns in interpreting these findings that must be considered are: *First*, it is essential to note that although patients received thorough training in using the HB-tDCS device, we did not remotely monitor their sessions, but the researcher was available to solve any problem with the device at any time. One must be cautious when comparing directly with studies that employed electrode placement and exposure supervision. *Second*, as recorded by the device software, the adherence rate is consistent with past research that supported this method of self-application for prolonged HB-tDCS use at home [12, 13]. *Third*, the HB-tDCS system utilized in this study offers an efficient technical solution that allows medical engineers who were not directly involved in the patient’s care to program the device according to the randomization sequence. This approach ensures the blinding of the research team and patients, eliminating bias in the study design. *Fourth*, it is essential to note that even though the randomization process ensured equal distribution between the groups that received HB-a-tDCS and HB-s-tDCS, other factors may still affect electrophysiological signals. These factors encompass psychiatric comorbidities like depression, anxiety, and sleep disturbances alongside medication use, particularly opioids, which can alter cortical reactions. Moreover, side effects, which are challenging to control completely, should be considered [80]. *Fifth*, we chose a 2:1 randomization ratio for practical reasons. This decision aimed to maximize the number of participants receiving active intervention, enhancing the exploration of potential benefits. Additionally, a higher chance of receiving active treatment may increase participant retention, particularly when the placebo may not offer immediate benefits. This strategy aimed to improve participant engagement and trial adherence over time. Ethically, prioritizing active treatment recipients aligns with addressing the substantial suffering of FM, offering more individuals a chance to alleviate their symptoms potentially [55, 81]. *Sixth*, our study revealed a high adherence rate, exceeding 85% for HB-a-tDCS and HB-s-tDCS sessions. We employed a rigorous and replicable methodology to showcase the effectiveness and practicality of HB-tDCS. *Seventh*, even though we used a standardized data collection protocol and our analysis showed that we had enough statistical power for the primary outcomes, we decided to exclude five patients in order to prioritize the quality of EEG signals and improve internal validity. Interpreting the results of this multi-group study requires caution, as we anticipate a high variability of EEG measures coupled with our small sample size, which could lead to Type II errors for secondary outcomes. *Eighth*, even though using 18 EEG electrodes for source localization may mean that we do not fully capture the intricate details of brain activity across different regions, our results are still beneficial for understanding how neural pain is processed and affects symptoms. This pragmatic electrode placement approach balances practical considerations with spatial resolution [36, 82]. Numerous studies have supported our strategy, including those on fibromyalgia (FM), obsessive-compulsive disorder (OCD), tinnitus, and autism spectrum disorder (ASD) [83–85]. Finally, to mitigate potential bias related to sex, we included only females, considering the estimated prevalence of fibromyalgia being 5:1 in women compared to men. Besides, previous research indicates that active tDCS over the DLPFC results in a greater flow of current toward the frontal regions in women [86]. Additionally, other studies suggest that women respond better to active tDCS than men [87].

## 5. Conclusion

These effects on these frequency bands may serve as potential neurophysiological markers, providing a deeper understanding of the impact of HB-a-tDCS on alleviating clinical symptoms of fibromyalgia. These findings add a neurophysiological basis to support the growing body of evidence for the clinical impact of twenty sessions of HB-tDCS over four weeks, targeting either the left DLPFC or the HB-a-tDCS over the M1.

## Supporting information

S1 File CONSORT checklist. (DOCX)

S2 File Checklist to use tDCS at home protocol. (DOCX)

S3 File Protocol and ethical approval. (PDF)

S4 File EEG resting-state protocol. (PDF)

S1 Table Supporting data. This is the S1 Table legend: We did not find a significant effect size for FIQ before to after HB-tDCS treatment in the active and sham tDCS over DLPFC groups. The HB-a-tDCS over the M1 group demonstrated a moderate effect size, *d* = 0.51. The HB-s-tDCS over the M1 group was not significant. The NPS difference in the active and sham HB-tDCS over DLPFC groups had a large effect size before to after HB-tDCS treatment, *d* = 1.03 and *d* = 0.91, respectively. The HB-a-tDCS over M1 group exhibited a moderate effect size, *d* = 0.42, and the HB-s-tDCS over the M1 group was not significant. The BDI before to after HB-a-tDCS treatment over DLPFC produce a small effect size, *d* = 0.38. The HB-s-tDCS group showed a large effect size, *d* = 0.96. In the tDCS stimulation over M1, the active group presented a moderate effect size *d* = 0.76, whereas the sham group was not significant. The effect size calculated for BDNF levels before to after HB-tDCS treatment in the HB-a-tDCS over DLPFC group showed a moderate effect size, *d* = 0.57. For the HB-s-tDCS over DLPFC group, the HB-a-tDCS over M1 group, and the HB-s-tDCS over M1 group, the effect size was not significant.
(PDF)
