# Supplementary material for: Modulation of neural networks and symptom correlated in fibromyalgia: A randomized double-blind multi-group explanatory clinical trial of home-based transcranial direct current stimulation
Source: PLoS One. 2024 Nov 13;19(11):e0288830. doi: 10.1371/journal.pone.0288830 (PMC11560039; doi:10.1371/journal.pone.0288830)
Supplement: S2 File — (DOCX) [file pone.0288830.s002.docx]

**Home Use tDCS Protocol**

**Pain and Neuromodulation Laboratory of the Hospital de Clínicas de Porto Alegre and the Federal University of Rio Grande do Sul - Brazil*.***

*E-mail: wcaumo@hcpa.edu.br*

Details about the device can be found at the following link: ([*https://www.jove.com/t/57614/home-based-transcranial-direct-current-stimulation-device-development*](about:blank)*) Carvalho, F., Brietzke, A.P., Gasparin, A., dos Santos, F.P., Vercelino, R., Ballester, R.F., Sanches, P.R., da Silva Jr, D.P., Torres, I.L., Fregni, F., Caumo, W. Home-Based Transcranial Direct Current Stimulation Device Development: An Updated Protocol Used at Home in Healthy Subjects and Fibromyalgia Patients. J. Vis. Exp. (137), e57614, doi:10.3791/57614 (2018).*

Instructions for patients can be viewed at the following link: [*https://youtu.be/3Wtji4esOGE*](about:blank)

1. *Preparation of materials for the First Visit*
   1. Prepare the material kit for each participant, including neoprene cap, tDCS device, battery charger, 2 electrodes, 2 silicone cannulas attached to the electrodes, 2 syringes containing 10ml of saline solution each, and 2 additional empty syringes, 2 bottles of saline solution. How to choose the cap size for each participant: Measure the circumference of the head and choose the cap size accordingly. There are three cap sizes available: small (38cm x 55cm), medium (39cm x 57.5cm), large (40cm x 59cm). The cap has a Velcro strap that allows for adjustment to the head. The patient should wear the cap, and the contact of the sponges with the scalp should be checked. The cap should not be too tight or too loose, but comfortable. I suggest including the figures from the manual or referring to the figures in the manual.
   2. Place the cap on the participant's head and measure the location for proper electrode placement according to the 10-20 EEG system.
   3. Position the sponges according to the target area (e.g., primary motor cortex, prefrontal cortex, etc.).
   4. Remove the cap from the participant's head to perform the perforation of the F3 and F4 positions according to the 10-20 EEG system and then attach the electrodes.
   5. Insert the 35cm2 electrodes into the sponge to deliver the electrical current to the scalp.
   6. Connect the silicone cannulas to the metal part to allow for saline solution infusion.
   7. Connect the electrode cable to the tDCS device (red to anode and black to cathode).
2. *tDCS Training Session*
   1. Inspect the scalp for the presence of any injuries or lesions that contraindicate tDCS (e.g., skin erythema, cuts, abrasions, etc.).
   2. With the participant in front of a mirror, instruct them to part their hair and expose the area to be stimulated.
   3. Clean the scalp with an alcohol-soaked cotton pad to remove creams, dirt, or oiliness.
   4. After the participant puts on the cap, instruct them that the seam may be at the level and between the eyebrows.
   5. Connect the syringes containing saline solution to the silicone cannulas (use approximately 6ml for each sponge).
   6. Conduct a training session on how to use the equipment in person.
   7. The first treatment session should be done under direct supervision immediately after the completion of the training session.
3. *Sequence of Screens*
   1. Turn on the device, and the first screen will appear: Opening Screen.
   2. Wait a few seconds until the second screen appears, which will show the Battery Status.
   3. Sequentially, the third screen will display the date and time.
   4. The next screen will give the command for the participant to wear the cap. After ensuring that the cap is properly adjusted, instruct them to press the center button.
   5. Wait for the command to inject saline solution. Ensure that the saline is injected slowly using the attached syringe. Inject 6ml of saline into each electrode and then press the center button.
4. *The sixth screen is the command to start the session*
   1. The participant should only press the central button when ready to begin stimulation.
   2. At the appearance of the seventh screen, indicating the start of stimulation, the following information will be displayed: session duration, battery status, and two lines. The top line shows the intensity (I), and the bottom line shows the resistance (R).

NOTE: The intensity will gradually increase until it reaches 2mA (or another predefined intensity). The resistance should reach approximately half the length of this line (5kΩ), and the investigator should set the device to the maximum impedance limit required.

1. Wait for the end of the stimulation, and after 20 minutes (or another predetermined time), the tDCS device will turn off, ceasing the stimulation. Session data will be automatically saved in the device software.

NOTE: The device saves each session with the date and time of stimulation, resistance, and total duration of the procedure.

1. *Safety*

If the participant reports discomfort after the start of stimulation, moisten the sponges with saline solution to reduce symptoms.

- 1. Pay attention to the presence of high resistance. In the case of high resistance, the device will sound an alarm instructing to "adjust the cap and inject more saline." At this moment, readjust the electrodes on the head and proceed with the injection of additional saline, not exceeding 10ml per electrode.
  2. Interrupt the session if necessary. The central button should be held down for a few seconds, followed by pressing the power button. A message "Turn off?" will appear.
  3. Do not repeat the session within 24 hours. If the last session lasted less than 50% of the total time, it is possible to repeat the session on the same day. However, if the last session lasted more than 50% of the intended time, it will not be possible to replicate the stimulation since the initial session will be considered valid.
  4. Charge the battery. The device has a rechargeable 9V battery. When the battery level reaches 50%, it will need to be recharged.
  5. To do this, disconnect the electrode cable and replace it with the charger cable. It is not possible to recharge the device during a stimulation session.

1. Home tDCS Sessions
   1. Instruct the patient to choose a calm place and time in their daily routine to perform the session. There should be no interruptions or pauses during the session. The activity should be guided according to the study protocol and followed in the same way for all sessions.
   2. The cap has an adjustable Velcro strap at chin level, allowing the cap to stay in place during stimulation. Additionally, the cap has a second Velcro strap in the frontal region, allowing better adjustment and contact of the electrodes with the scalp.
   3. The first home session corresponds to the second treatment session and is performed at the participant's home. The first home treatment session is conducted under the supervision of a research team member via video call. In subsequent sessions, the team remains available for any questions.
   4. During the stimulation session:
   5. Participants will be informed that they may feel a slight tingling or itching sensation at the site where the electrodes are positioned.
   6. After session remove the cap and place it in a well-ventilated area to dry so that it can be used in the next session.
   7. During the designated treatment period, remove the electrodes for cleaning and wash the sponges at least once a week.
2. *Procedures for washing:*
   1. Attach the sponges to the snap button inside the cap. The buttons are fastened as described above (see: Item 1. How to choose the cap size for each participant). After placing the clean sponges, insert the electrodes into them. The process is done under the supervision of a researcher via video call once a week.
3. *Adverse Effects*
   1. Instruct the participant to correctly fill out the daily adverse effects questionnaire immediately after each home session.
   2. Provide each participant with all the information in printed form and a telephone contact. This phone number will be available 24 hours for any questions or issues related to using the device. Participants should be able to contact the research team at any time.
   3. Ask the participant about the approximate time the sessions will be conducted so that the team can be aware of any potential increased demands.

NOTE: After completing the protocol, the participant should complete the final evaluation and return the tDCS device and the adverse effects diary.

1. *Access to Software and Information*
   1. Access to the recorded information should be done at the end of the treatment. To do this, use a computer and cable to extract the data saved in the software. The data will show all the sessions conducted and provide the values of current intensity and impedance for each session. Participants will not have access to this information.
2. *Availability of the Research Team*
   1. Provide each participant with all the information in printed form and a telephone contact. This phone number will be available 24 hours for any questions or issues related to using the device. Participants should be able to contact the research team at any time.
   2. Ask the participant about the approximate time the sessions will be conducted so that the team can be aware of any potential increased demands.

NOTE: After completing the protocol, the participant should complete the final evaluation and return the tDCS device and the adverse effects diary.

1. *Access to Software and Information*
   1. Access to the recorded information should be done at the end of the treatment. To do this, use a computer and cable to extract the data saved in the software. The data will show all the sessions conducted and provide the values of current intensity and impedance for each session. Participants will not have access to this information.
