# Supplementary material for: Modulation of neural networks and symptom correlated in fibromyalgia: A randomized double-blind multi-group explanatory clinical trial of home-based transcranial direct current stimulation
Source: PLoS One. 2024 Nov 13;19(11):e0288830. doi: 10.1371/journal.pone.0288830 (PMC11560039; doi:10.1371/journal.pone.0288830)
Supplement: S3 File — (PDF) [file pone.0288830.s003.pdf]

FACULDADE DE MEDICINA DA UFRGS

HOSPITAL DE CLÍNICAS DE PORTO ALEGRE (HCPA)

LABORATÓRIO DE DOR E NEUROMODULAÇÃO DO HCPA

UNIVERSIDADE FEDERAL DO RIO GRANDE DO SUL (UFRGS)

TÍTULO: ***MAPEAMENTO DO IMPACTO DA MONTAGEM DA ESTIMULAÇÃO TRANSCRANIANA DE CORRENTE CONTÍNUA DOMICILIAR NAS FUNÇÕES CORTICAIS, PSICO-COGNITIVAS, CAPACIDADE FUNCIONAL E RITMO BIOLÓGICO NA FIBROMIALGIA: UM ENSAIO CLÍNICO RANDOMIZADO FATORIAL (ACRÔNIMO: HFTDCS-TRIAL)***

Coordenador: Professor Wolnei Caumo, MD., PhD.

Professor Titular do Departamento de Cirurgia da Faculdade de Medicina da Universidade Federal do Rio Grande do Sul (UFRGS). Chefe do Serviço de Dor e Medicina Paliativa do HCPA. Chefe do Laboratório de Dor e Neuromodulação do HCPA.

Membros da equipe:

Maxciel Zortea, Psy, PhD. Pesquisador associado do Laboratório de Dor e Neuromodulação do HCPA.

Leticia Ramalho, RD, PhD. Pesquisador associado do Laboratório de Dor e Neuromodulação do HCPA.

Camila Fernanda da Silveira Alves, PhD. Pesquisador associado do Laboratório de Dor e Neuromodulação do HCPA.

Rael Lopes Alves– doutorando do Programa de Pós-graduação em Medicina Ciências Médicas da FAMED- UFRGS.

Paul Vicunha - doutorando do Programa de Pós-graduação em Medicina Ciências Médicas da FAMED- UFRGS.

Vani Laranjeira - doutoranda do Programa de Pós-graduação em Medicina Ciências Médicas da FAMED- UFRGS.

Samuel Lopes Souza - Bolsista de iniciação científica e acadêmico de Medicina.

Álvaro Franco - Bolsista de iniciação científica e acadêmico de Medicina.

School of Medicine UFRGS
Clinics Hospital of Porto Alegre
Laboratory of Pain and Neuromodulation
Federal University of Rio Grande do Sul

TITLE: MAPPING THE IMPACT OF THE STIMULATION TRANSCRANIAL DIRECT CURRENT AT HOME ASSEMBLY ON CORTICAL FUNCTIONS, PSYCHO-COGNITIVE, FUNCTIONAL CAPACITY, AND BIOLOGICAL RHYTHM IN FIBROMYALGIA: A FACTORIAL RANDOMIZED CLINICAL TRIAL (ACRONYM: HFTDCS-TRIAL)

Coordinator: Professor Wolnei Caumo, MD., PhD.
Professor of the Department of Surgery of the School of Medicine, UFRGS. Principal of Pain and Palliative Care Service at HCPA; Principal of the Laboratory of Pain and Neuromodulation at Hospital de Clínicas de Porto Alegre (HCPA):

Staff Members:
Maxciel Zortea, Psy, PhD. Researcher associated to Laboratory of Pain and Neuromodulation (HCPA),
Leticia Ramalho, RD, PhD. Researcher associated to Laboratory of Pain and Neuromodulation (HCPA),
Camila Fernanda da Silveira Alves, PhD. Researcher associated to Laboratory of Pain and Neuromodulation (HCPA),
Rael Lopes Alves – Post-Graduate Program in Medical Sciences, School of Medicine, UFRGS.
Paul Vicunha - Post-Graduate Program in Medical Sciences, School of Medicine, UFRGS.
Vani Laranjeira - Post-Graduate Program in Medical Sciences, School of Medicine, UFRGS.
Samuel Lopes Souza - scientific initiation scholarship holder, medicine student.
Álvaro Franco - scientific initiation scholarship holder, medicine student.

econômico ao paciente e à sociedade

3 OBJETIVOS

3.1 OBJETIVO GERAL

Mapear o impacto da ETCC domiciliar prolongada aplicada no córtex motor primário e no córtex pré-frontal dorsolateral esquerdo, comparadas aos respectivos tratamentos simulados, tendo como desfechos marcadores neurofisiológicos, aspectos psico-cognitivos, capacidade funcional e ritmo biológico na fibromialgia.

3.2 OBJETIVOS ESPECÍFICOS

Este estudo visa mapear o impacto da ETCC anódica de uso domiciliar prolongado aplicada no córtex motor primário e no córtex pré-frontal dorsolateral esquerdo, comparadas aos respectivos tratamentos simulados na fibromialgia, tendo como alvo os desfechos dos seguintes eixos: (1) intensidade da dor e capacidade funcional; (2) funções psicocognitivas; (3) marcadores neurofisiológicos; (4) marcadores do ritmo biológico; (5) mapear o potencial dos marcadores neurofisiológicos, de ritmicidade biológica e história de trauma na infância e adolescência na capacidade funcional, sintomas depressivos e testes de memória de trabalho.

1. Desfechos relacionados à intensidade da dor e capacidade funcional:

   1.1. Capacidade funcional para atividades da vida diária avaliada pelo Questionário de Impacto da Fibromialgia (QIF) e Escala Funcional de Dor (desfecho primário);

   1.2. Níveis de dor registrado no diário da Escala Análogo-Visual de Dor nas últimas 24h, assim como o consumo de analgésicos;

   1.3. Limiar de dor ao calor e pressão, somação temporal e a função do sistema modulatório descendente de dor por meio da mudança no escore da Escala Numérica de dor (NPS 0-10) ao teste de modulação condicionante da dor (CPM);

2. Desfechos relacionados a funções psicocognitivas:

   2.1. Capacidade de recuperação da memória episódica por meio do índice de recuperação do Teste de aprendizagem auditivo-verbal de Rey (RAVLT) (desfecho primário);

   2.3. Recuperação imediata e reconhecimento de memória episódica;

   2.4. Memória de curto prazo e memória de Trabalho (span de dígitos e índice de

Pag 2. Item 3 of the Project submitted to Ethic Commite

3 AIMS

3.1 General Objective:

Mapping the impact of extended tDCS at home applied on the primary motor cortex and on the left dorsolateral prefrontal cortex, compared to the respective simulated treatments, having as outcomes neurophysiological markers, psycho-cognitive aspects, functional capacity and biological rhythm in fibromyalgia.

3.2 Specific goals:

This study aims to map the impact of anodic home -based tDCS for prolonged use applied in the primary motor cortex and in the left dorsolateral prefrontal cortex, compared to the respective simulated treatments in fibromyalgia, targeting the outcomes of the following axes: (1) pain intensity and functional capacity; (2) psychocognitive functions; (3) neurophysiological markers; (4) biological rhythm markers; (5) map the neurophysiological potential markers, biological rhythmicity, and history of trauma in childhood and/or adolescence on functional capacity, depressive symptoms and working memory tests.

1. Outcomes related to pain intensity and functional capacity:
   1.1. Functional capacity for activities of daily living assessed by Fibromyalgia Impact Questionnaire (FIQ) and Functional Pain Scale (primary outcome);
   1.2. Pain levels recorded in the Numeric Pain Scale diary in the last 24 hours, as well as the consumption of analgesics;
   1.3. Heat and pressure pain threshold, temporal summation and the function of the modulatory system descending pain through change in Numerical Pain Scale score (NPS 0-10) the pain conditioning modulation test (CPM);

2. Outcomes related to psychocognitive functions:
   2.1. Episodic memory retrieval through index of retrieval the Rey Auditory-Verbal Learning Test (RAVLT) (primary outcome);
   2.3. Immediate retrieval and recognition of episodic memory;
   2.4. Short-term memory and working memory (digit span and discrimination index);

discriminação);

3. Desfechos relacionados aos marcadores neurofisiológicos:

3.1 Níveis de conectividade (intra- e inter-sujeitos) baseada na análise de correlação de sementes de áreas alvos no processamento da dor como: DLPFC, M1 tendo como parâmetro os níveis de oxi (HbO2) e desoxihemoglobina (HbR) em repouso e após o cold pressure test (desfecho primário);

3.2. Sinal eletrofisiológico do cérebro obtido pelo eletroencefalograma (EEG, poder de onda) em estado de repouso;

3.3. Medidas de excitabilidade cortical obtidas pela EMT (período silente e potencial evocado motor);

4. Desfechos relacionados aos marcadores do ritmo biológico (primário: ritmo secretório da aMT6s e a eficiência do sono).

4.1. Parâmetros relacionados à qualidade do sono mensurados pela actimetria, tais como a variabilidade intradiária, período dos despertares noturnos após o início do sono, latência para iniciar o sono e o tempo total de sono.

4.2. Ritmos de temperatura e luminosidade avaliadas por meio da actigrafia.

4.3. Qualidade do sono avaliada pelo diário de sono e pelo Questionário de Pittsburgh.

4.4. Ritmo secretório da 6-sulfatoximelatonina urinaria (aMT6s) no curso das 24h.

5. Explorar o potencial preditivo, nos desfechos relacionados à capacidade funcional devido a dor sintomas depressivos e desempenho nos testes de memória de trabalho dos seguintes parâmetros:

5.1 Nível de inibição intracortical e potencial evocado motor;

5.2 Níveis de BDNF, S-100B e genótipos do polimorfismo rs6265 do gene do *BDNF*;

5.3 Nível de conectividade inter-hemisférica do DLPFC e M1;

5.4 Ritmo secretório da 6-sulfatoximelatonina urinaria (aMT6s) no curso das 24h;

3. Outcomes related to neurophysiological markers:

3.1 Levels of connectivity (intra- and inter-subjects) based on the correlation analysis of seeds of target areas in pain processing such as: DLPFC, M1, having as a parameter the levels of oxy (HbO2) and deoxyhemoglobin (HbR) at rest and after the cold pressure test (primary outcome);

3.2. Electrophysiological signal of the brain obtained by electroencephalogram (EEG, wave power) in resting state;

3.3. Cortical excitability measurements obtained by TMS (silent period and motor evoked potential);

4. Outcomes related to biological rhythm markers (primary: aMT6s secretory rhythm and sleep efficiency).

4.1. Parameters related to sleep quality measured by actigraphy, such as intraday variability, period of nocturnal awakenings after sleep onset, latency to sleep onset and total sleep time.

4.2. Temperature and luminosity rhythms evaluated by means of actigraphy.

4.3. Sleep quality assessed using the sleep diary and the Pittsburgh Sleep Quality Inventory (PSQI).

4.4. Secretory rate of urinary 6-sulphatoxymelatonin (aMT6s) over 24 hours.

5. Explore the predictive potential, in outcomes related to functional capacity due to pain, depressive symptoms and performance in working memory tests of the following parameters:

5.1 Level of intracortical inhibition and motor evoked potential (MEP);

5.2 Levels of BDNF, S-100B and genotypes of the BDNF gene rs6265 polymorphism;

5.3 Level of inter-hemispheric connectivity of the DLPFC and M1;

5.4 Secretory rhythm of urinary 6-sulphatoxymelatonin (aMT6s) over 24 hours;

8. SEQUÊNCIA EXPERIMENTAL

A sequência experimental na linha de base está apresentada na **Figura 7** com o fluxograma do estudo. No ambiente domiciliar as pacientes que realizarão as sessões de ETCC ativa ou simulada e serão orientadas para fazerem exercícios. O tipo e o tempo de exercícios realizado serão avaliados por meio de um diário.

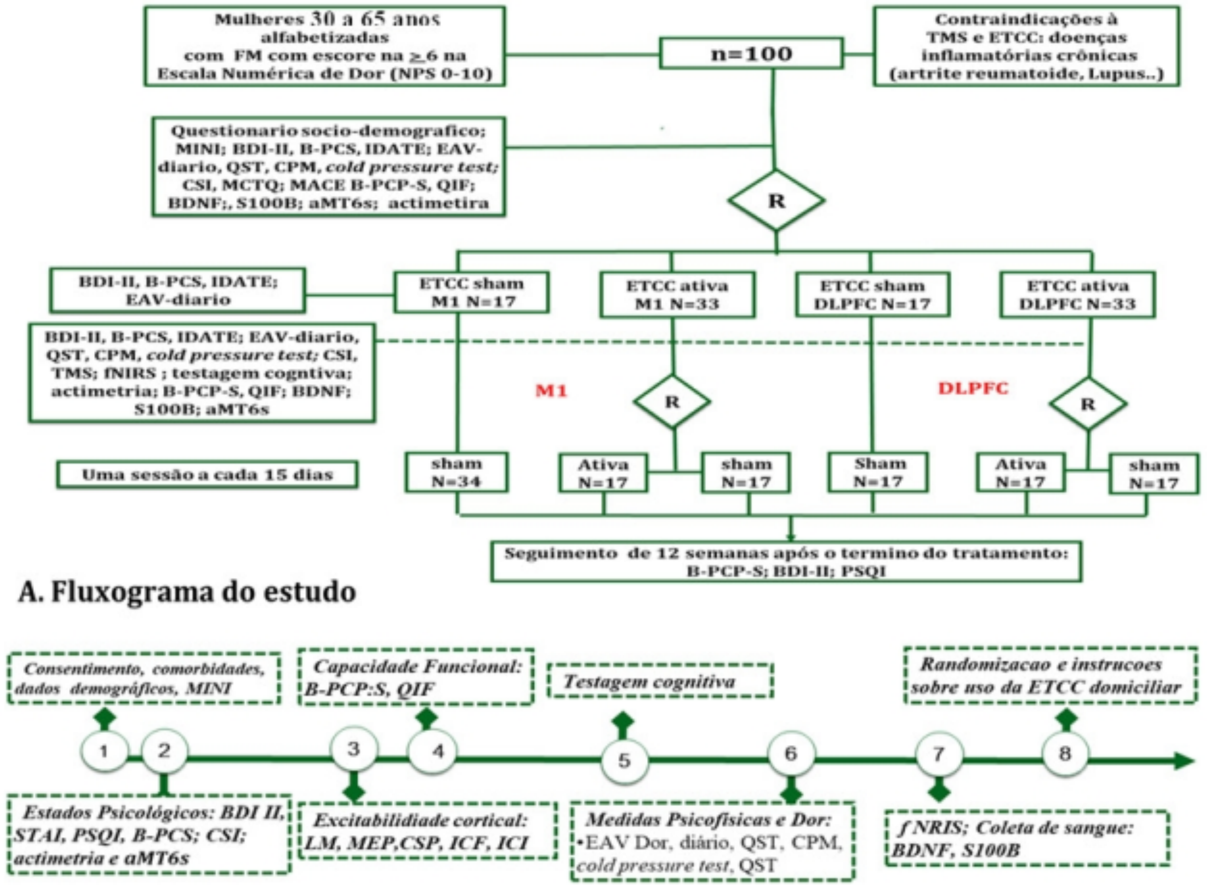

Figura 7. Avaliação da linha de base: Diário de dor, consumo de analgésicos e efeitos adversos serão aferidos diariamente até o final do tratamento 8ª semana. Questionário de Sono de Pittsburgh (Bertolazi et al., 2011). MINI Structured Clinical Interview for DSM-IV Axis I Disorders (SCID-I) (Spitzer et al. 1988); International Neuropsychiatric Interview (MINI), actimetria e diário de sono durante 10 dias. Munich ChronoType Questionnaire (MCTQ). **Sequência experimental: Sensibilização central:** Inventário de Sensibilização Central (CSI) e somação temporal avaliada por meio do cold pressor test e potencial evocado somatossensitivo (PESS). Inventário de Sensibilização Central (ICS) (Caumo, et al. 2017). **Avaliação do estado emocional:** Inventário de Depressão (BDI-II) (Gorenstein et al., 2011), Escala de Catastrofismo sobre Dor - para uso no Brasil (B-PCS) (Sehn et al., 2012), **Medidas psicofísicas e avaliação de dor** - Quantitative Sensory-Testing (QST), clod pressor test (CPT); Teste de modulação condicionada da dor (CPM), Escala Análogo Visual de Dor (EAV). **Capacidade funcional:** Screening para avaliar o impacto funcional da dor crônica - Brazilian Profile of Chronic Pain Screen (B-PCP:S) (Caumo et al., 2013), Questionário de Impacto de Fibromialgia (QIF) (Marques et al., 2006). **Conectividade funcional cerebral:** Ressonância magnética funcional (fMRI), EEG, fNIRS. **Excitabilidade cortical:** Potencial evocado motor (MEP), inibição intracortical (ICI), facilitação intracortical (ICF), período silente (CSP). **Marcadores séricos de neuroplasticidade:** Brain-Derived-Neural Factor (BDNF), proteina S100-B. Dosagem dos níveis urinários de 6-sulfatoximelatonina (MT6s).

8. EXPERIMENTAL SEQUENCE

The baseline experimental sequence is shown in Figure 7 with the flowchart of the study. In the home environment, patients who will undergo active or simulated tDCS sessions and will be oriented to exercise. The type and duration of exercises performed will be evaluated through a diary.

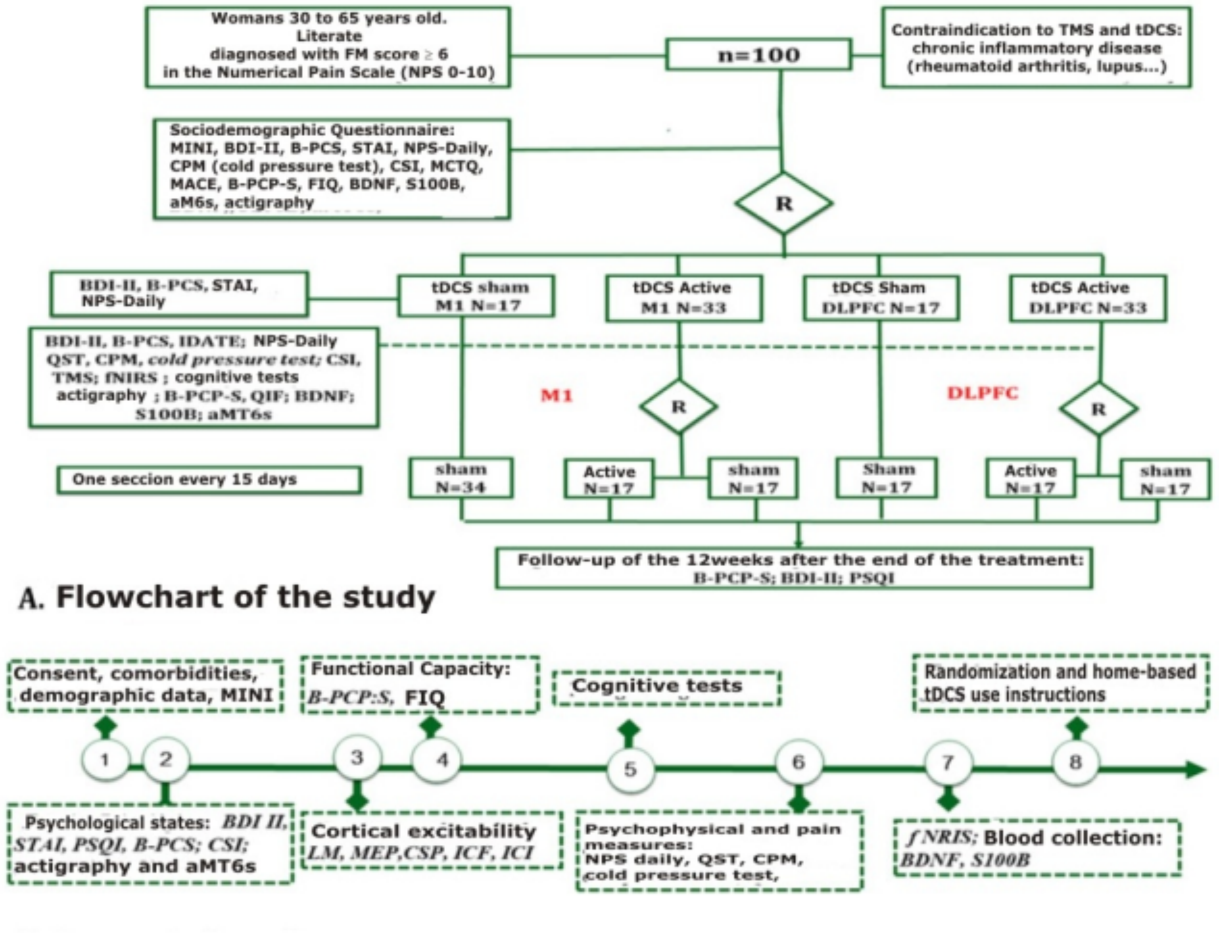

Figure 7. Baseline assessment: Pain diary, analgesic consumption and adverse effects will be measured daily until the end of treatment 8th week. Pittsburgh Sleep Quality Inventory (Bertolazi et al., 2011). MINI Structured Clinical Interview for DSM-IV Axis I Disorders (SCID-I) (Spitzer et al. 1988); International Neuropsychiatric Interview (MINI), actigraphy and sleep diary for 10 days. Munich ChronoType Questionnaire (MCTQ). Experimental sequence: Central Sensitization: Central Sensitization Inventory (CSI) and temporal summation assessed using the cold pressor test and somatosensory evoked potential (SEP). Central Sensitization Inventory (CSI) (Caumo, et al. 2017). Emotional state assessment: Depression Inventory (BDI-II) (Gorenstein et al., 2011), Pain Catastrophizing Scale - for use in Brazil (B-PCS) (Sehn et al., 2012), Psychophysical measures and assessment of pain - Quantitative Sensory-Testing (QST), cloud pressor test (CPT); Conditioned Pain Modulation Test (CPM), Numerical Pain Scale (NPS). Functional capacity: Screening to assess the functional impact of chronic pain - Brazilian Profile of Chronic Pain Screen (B-PCP:S) (Caumo et al., 2013), Fibromyalgia Impact Questionnaire (FIQ) (Marques et al., 2006). Functional brain connectivity: Functional magnetic resonance imaging (fMRI), EEG, fNIRS. Cortical excitability: Motor evoked potential (MEP), intracortical inhibition (ICI), intracortical facilitation (ICF), silent period (CSP). Serum neuroplasticity markers: Brain-Derived-Neural Factor (BDNF), S100-B protein. Measurement of urinary levels of 6-sulphatoxymelatonin (MT6s).

HOSPITAL DE CLÍNICAS DE PORTO ALEGRE

Grupo de Pesquisa e Pós Graduação

Carta de Aprovação

Projeto

2020/0369

Pesquisadores:

WOLNEI CAUMO

LETICIA RAMALHO	CAMILA FERNANDA DA SILVEIRA ALVES	VANI DOS SANTOS LARANJEIRA
PAUL SERRANO VICUÑA	SAMUEL LOPES SOUZA	MAXCIEL ZORTEA
RAEL LOPES ALVES	ALVARO DE OLIVEIRA FRANCO	

Número de Participantes: 102

Título: MAPEAMENTO DO IMPACTO DA MONTAGEM DA ESTIMULAÇÃO TRANSCRANIANA DE CORRENTE CONTÍNUA DOMICILIAR NAS FUNÇÕES CORTICAIS, PSICOCOGNITIVAS, CAPACIDADE FUNCIONAL E RITMO BIOLÓGICO NA FIBROMIALGIA: UM ENSAIO CLÍNICO RANDOMIZADO FATORIAL (ACRÔNIMO: HFTDCS-TRIAL)

Este projeto foi APROVADO em seus aspectos éticos, metodológicos, logísticos e financeiros para ser realizado no Hospital de Clínicas de Porto Alegre.
Esta aprovação está baseada nos pareceres dos respectivos Comitês de Ética e do Serviço de Gestão em Pesquisa.

- Os pesquisadores vinculados ao projeto não participaram de qualquer etapa do processo de avalição de seus projetos.

- O pesquisador deverá apresentar relatórios semestrais de acompanhamento e relatório final ao Grupo de Pesquisa e Pós-Graduação (GPPG).

04/12/2020

Assinado digitalmente por:
PATRICIA ASHTON PROLLA
Grupo de Pesquisa e Pós-graduação
07/12/2020 15:48:10
https://aghuse-externo.hcpa.edu.br/pesquisa/publico/cadastroapoio/conferenciaArquivo.xhtml?co

Clinics Hospital of Porto Alegre
Research and Postgraduate Group

Approval Letter

Project
2020/0369

Researchers:
WOLNEI CAUMO

Leticia Ramalho	Camila Fernanda da Silveira Alves	Vani dos Santos Laranjeira
Paul Serrano Vicuña	Samuel Lopes Souza	Maxciel Zortea
Rael Lopes Alves	Alvaro de Oliveira Franco	

Number of Participants: 102

Title: Mapping the impact of the transcranial direct current stimulation at home assembly on cortical functions, psycho-cognitive, functional capacity, and biological rhythms in fibromyalgia: a factorial randomized clinical trial (Acronym: HFTDCS-TRIAL)

This project was APPROVED in its ethical, methodological, logistical, and financial aspects to be carried out at Clinics Hospital of Porto Alegre.
This approval is based on the counsel of the respective Ethics Committees and the Research Management Service.

- The researchers linked to the project didn't participate in any evaluation process stage of their projects.

- The researcher must submit semi-annual follow-up reports and a final report to the Research and Postgraduate Group.

04/12/2020
