# Supplementary material for: Modulation of neural networks and symptom correlated in fibromyalgia: A randomized double-blind multi-group explanatory clinical trial of home-based transcranial direct current stimulation
Source: PLoS One. 2024 Nov 13;19(11):e0288830. doi: 10.1371/journal.pone.0288830 (PMC11560039; doi:10.1371/journal.pone.0288830)
Supplement: S4 File — (PDF) [file pone.0288830.s004.pdf]

LABORATORY OF PAIN AND NEURMODULATION

Procedure and protocol to EEG resting-state

CORRESPONDING AUTHOR: *Wolnei Caumo MD, PhD; Department: Laboratory of Pain and Neuromodulation; Institution: Hospital de Clínicas de Porto Alegre at UFRGS. Address: Ramiro Barcelos, 2350 - CEP 90035-003 Bairro Rio Branco - Porto Alegre – RS. Phone: (55) 51-3359.8083. Fax: (55) 51- 3359.8083.E-mail: wcaumo@hcpa.edu.br*

1. DEVICE DESCRIPTION

The EEG system used was the ENOBIO 20, Neuroelectrics (Barcelona, SP):

- Number of channels: 18 scalp sites according to the 10–20 system (FP1, FP2, F7, F3, Fz, F4, F8, T7, C3, Cz, C4, T8, P7, P3, Pz, P4, P8, Oz).
- Earclip (left ear (EXT) / right ear (CMS/DRL)): It is a dual reference electrode because it is used to connect the two reference channels, CMS and DRL, to the same earlobe.
- Circular gel electrodes with a contact area of 1.75 $cm^{2.}$
- Impedance was 5 kΩ for all electrodes
- EEG functionality:
  - Sampling rate: 500 SPS
  - Bandwidth: 0 to 125 Hz (DC coupled)
  - Resolution: 24 bits - 0.05 µV
  - Measurement noise: < 1 µV RMS
  - Communication: Enobio is a wireless device. Wi-Fi iEEE 802.11 g or USB (Only available for use with USB Isolator accessory cable). The Necbox connects through Wi-Fi to the Neuroelectrics Instrument Controller (NIC) software running on a computer. The EEG data is streamed through the Wi-Fi band, which has an operating distance range of 10 meters or less.
    - Wireless Specifications:
      - Wi-Fi iEEE 802.11 g
      - Operating frequency band:2412 ~ 2472 MHz
      - Transmitting powerMax. 16 ~+17.6 dBm
  - Output: EDF+ (16 bits), ASCII data files or TCP/IP raw data streaming

2. EEG RESTING-STATE

Recent studies demonstrates that resting states EEG might be a potential tools to evaluated chronic pain conditions because presented an oscillatory brain signature, abnormal oscillations at theta, alpha and beta frequencies (Ploner, Sorg, Gross, 2017; Mussigmann, Bardel, Lefaucheur, 2022). We perform a EEG resting-state acquisition follow the pattern approach. The register were made in a quiet room. The subjects were oriented to sitting in a comfortable armchair, and keep their eyes on a black cross fixed on the front wall at your eye level, positioned 1.5 m ahead of the armchair, remained relaxed and at rest, thinking free and avoid any specific think. The resting state EEG were recorded during 8 minutes, switching 2 minutes between eyes open (EO) and eyes closed (EC) conditions, using 18 channels (FP1, FP2, F7, F3, Fz, F4, F8, T7, C3, Cz, C4, T8, P7, P3, Pz, P4, P8, Oz, plus EXT (left ear) and the two reference channels, CMS and DRL (right ear), according to 10-20 system. The register was performed using a sampling rate of 500 Hz, impedance of 5 kΩ for all electrodes, with high dynamic resolution (24 bits, 0.05uV). A line noise filter (60-Hz) was applied to remove main line artifacts from the EEG data.

Preliminary data analysis were conducted by blinding researchers in the prior data, which define 2 seconds free artifacts as the segmentation length (epochs). This length is commonly used in EEG clinical research (Jobert et al., 2012). The choice of EEG segment length is related to frequency resolution. The longer the period, the higher the resolution.

Artifacts might be generated by instrumental problems such as poor electrode contact surface (high impedance), a loop between electrodes due sweat or conductive material. Biological artifacts includes blinking eyes, ocular movements (saccadic eye movements, nystagmus), cardiac beat, head or face muscular tension, head movements. Also, preliminary data analysis is dedicated to controlling the subject's vigilance during EEG recording, avoiding segments of EEG rhythms showing signs of drowsiness (significant attenuation of alpha oscillation or slowing in the frequency alpha rhythms followed by increased theta rhythms). These artifacts should be rejected to quantitative data analysis (Babiloni et al, 2020).

3. PROCEDURES

The procedures were conducted with the subjects sitting in a comfortable armchair. We wore a comfortable and adjusted headcap according to the head circumference of the participants and used three different head sizes: small (52 cm), medium (55 cm), and large (58 cm). We mark in the scalp the vertex measurement for Cz placement (50% of the nasion-inion distance, and 50% of the auricular distance).

The 18 circular gel electrodes positioned in the headcap based on the 10-20 system are flexible electrodes that allow access to the scalp. The electrode gel is applied on the contact surface between the electrode and the scalp to decrease the impedance and improve the signal quality.

The system digitizes analogue EEG signals collected by the headcap with electrodes, amplifies them, and uses wi-fi wireless connectivity to transmit the EEG data to a host computer with the software to visualization of the signal. A line noise filter (60-Hz) was applied to remove main line artifacts from the EEG data.

3. RESTING-STATE RECORDING

- Recorded Time: 8 minutes (switched 2 minutes between eyes-open (EO) and eyes closed (EC).
- We keep the light off in the room and ensure no noise in the surrounding area.
- We test the computer markers for the moments of eyes open (EO) and closed (EC): buttons 1 and 2 on the keyboard.
- Patients instruction:

  "In this task you will remain relaxed and at rest, being able to thinking free, avoid anything specific. I will ask you to keep your eyes open for 2 minutes, and you should keep their eyes fixed in the black cross positioned on the front wall at eye level. Then I will ask you to close your eyes for another 2 minutes. Close your eyes lightly, without forcing. Then we will repeat again, with another 2 minutes of eyes open and another 2 minutes of eyes closed.

During the test with your eyes open, try to blink as little and as lightly as possible. Stay relaxed, without moving your head or facial muscles."

- We start recording.
    - We pressing number 1 on the keyboard to mark the beginning of eyes open and button number 2 to begin eyes closed. If any interval (EO, EC) suffers interference on any channel, or noise of the surrounding area, we repeat the recording of this interval and take note of the occurrences (this will be essential for pre-processing).
- Once done, we end recording.
- We save the data at convenient location.

Data availability: Any data related to the intervention and primary outcomes will be available upon request from Caumo (wcaumo@hcpa.edu.br) without any time restrictions.

COMPETING INTERESTS:
WC, National Council for Scientific and Technological Development - (CNPq), Brazil, ILST reported grants from National Council for Scientific and Technological Development, and Fundación de Amparo à Pesquisa do Estado do Rio Grande do Sul during the conduct of the study and PRSS grants from National Council for Scientific and Technological Development during the conduct of the study. PRSS reported grants from Financiadora de Estudos e Projetos during the conduct of the study; in addition, WC, ILST, PRSS had a patent for BR 20 2015 016450 0 licensed to Quark Medical. The tDCS device used in this study was developed and patented by PRSS, DPSJ, ILST, and WC, and these authors received royalties from Quark Medical (patent for BR 20 2015 016450 0 licensed to Quark Medical). DPSJ reported grants from Financiadora de Estudos e Projetos during the conduct of the study. No other competing interests were reported. This does not alter our adherence to PLOS ONE policies on sharing data and materials.

FUNDING:
The following provided support for this study: (i) Committee for the Development of Higher Education Personnel (CAPES) through the academic excellence program (PROEX) (grants doctorate scholarships to RLA, and PVS), and the post-graduation national program (PNPD) (grant to MZ, LR, and CFSA). (ii) National Council for Scientific and Technological Development (CNPq) (Grants no 420826/2018-1 and 19/2551-0000716-7 to WC). (iii) Postgraduate Research Group at the Hospital de Clínicas de Porto Alegre (FIPE-HCPA) to WC (project no. 2020-0369). (iv) Brazilian Innovation Agency (FINEP) to WC, and ILST (process no. 1245/13). (v) Foundation for the Support of Research at Rio Grande do Sul (FAPERGS) Ministry of Science and Technology. National Council for Scientific and Technological Development - (CNPq)/ Health Secretary of State of Rio Grande do Sul, Brazil (SEARS) n. 03/2017 (PPSUS) to WC (Grant no: 17/2551-0001). The funders had no role in study design, data collection and analysis, decision to publish, or preparation of the manuscript.

REFERENCES:

1. ENOBIO: Neuroeletrics User Manual. Neuroeletrics, Barcelona, SP, 2021.

2. Alves RL, Zortea M, Serrano PV, Brugnera Tomedi R, Pereira de Almeida R, Torres ILS, et al. High-beta oscillations at EEG resting state and hyperconnectivity of pain circuitry in fibromyalgia: an exploratory cross-sectional study. Front Neurosci. 2023 Nov 27;17:1233979. doi: 10.3389/fnins.2023.1233979. PMID: 38089976.

3. Babiloni C, Barry RJ, Başar E, Blinowska KJ, Cichocki A, Drinkenburg WHIM, Klimesch W, Knight RT, Lopes da Silva F, Nunez P, Oostenveld R, Jeong J, Pascual-Marqui R, Valdes-Sosa P, Hallett M. International Federation of Clinical Neurophysiology (IFCN) - EEG research workgroup: Recommendations on frequency and topographic analysis of resting state EEG rhythms. Part 1: Applications in clinical research studies. Clin Neurophysiol. 2020 Jan;131(1):285-307. doi: 10.1016/j.clinph.2019.06.234. Epub 2019 Sep 19. PMID: 31501011.

4. Jobert M, Wilson FJ, Ruigt GS, Brunovsky M, Prichep LS, Drinkenburg WH; IPEG Pharmaco-EEG Guidelines Committee. Guidelines for the recording and evaluation of pharmaco-EEG data in man: the International Pharmaco-EEG Society (IPEG). Neuropsychobiology. 2012;66(4):201-20. doi: 10.1159/000343478. Epub 2012 Oct 12. PMID: 23075830.

5. Mussigmann T, Bardel B, Lefaucheur JP. Resting-state electroencephalography (EEG) biomarkers of chronic neuropathic pain. A systematic review. Neuroimage. 2022 Sep;258:119351. doi: 10.1016/j.neuroimage.2022.119351. Epub 2022 Jun 2. PMID: 35659993.

6. Ploner M, Sorg C, Gross J. Brain Rhythms of Pain. Trends Cogn Sci. 2017 Feb;21(2):100-110. doi: 10.1016/j.tics.2016.12.001. Epub 2016 Dec 23. PMID: 28025007; PMCID: PMC5374269.

7. Schwertner, A., Zortea, M., Torres, F. V., Ramalho, L., Alves, C. F. D. S., Lannig, G., et al.. S-ketamine's effect changes the cortical electrophysiological activity related to semantic affective dimension of pain: A placebo-controlled study in healthy male individuals. Frontiers in Neurosci. 2019 Sep 13. https://doi.org/10.3389/fnins.2019.00959

8. Schwertner, A., Zortea, M., Torres, F. V., & Caumo, W. Effects of subanesthetic ketamine administration on visual and auditory event-related potentials (ERP) in humans: A systematic review. Frontiers in Behavioral Neurosc. 2018; 12. https://doi.org/10.3389/fnbeh.2018.00070

9. Zortea M, Beltran G, Alves RL, Vicuña P, Torres ILS, Fregni F, et al. Spectral Power Density analysis of the resting-state as a marker of the central effects of opioid use in fibromyalgia. Sci Rep. 2021 Nov 22;11(1):22716. doi: 10.1038/s41598-021-01982-0. PMID: 34811404; PMCID: PMC8608932.
