# Supplementary material for: Modulation of neural networks and symptom correlated in fibromyalgia: A randomized double-blind multi-group explanatory clinical trial of home-based transcranial direct current stimulation
Source: PLoS One. 2024 Nov 13;19(11):e0288830. doi: 10.1371/journal.pone.0288830 (PMC11560039; doi:10.1371/journal.pone.0288830)
Supplement: S1 Table — This is the S1 Table legend: We did not find a significant effect size for FIQ before to after HB-tDCS treatment in the active and sham tDCS over DLPFC groups. The HB-a-tDCS over the M1 group demonstrated a moderate effect size, d = 0.51. The HB-s-tDCS over the M1 group was not significant. The NPS difference in the active and sham HB-tDCS over DLPFC groups had a large effect size before to after HB-tDCS treatment, d = 1.03 and d = 0.91, respectively. The HB-a-tDCS over M1 group exhibited a moderate effect size, d = 0.42, and the HB-s-tDCS over the M1 group was not significant. The BDI before to after HB-a-tDCS treatment over DLPFC produce a small effect size, d = 0.38. The HB-s-tDCS group showed a large effect size, d = 0.96. In the tDCS stimulation over M1, the active group presented a moderate effect size d = 0.76, whereas the sham group was not significant. The effect size calculated for BDNF levels before to after HB-tDCS treatment in the HB-a-tDCS over DLPFC group showed a moderate effect size, d = 0.57. For the HB-s-tDCS over DLPFC group, the HB-a-tDCS over M1 group, and the HB-s-tDCS over M1 group, the effect size was not significant. (PDF) [file pone.0288830.s005.pdf]

GROUPS: 1) a-tDCS over DLPFC 2) s-tDCS over DLPFC 3) a-tDCS over M1 4) s-tDCS over M1

DELTA= (PRE-POST)

1= YES
2= NO

CONNECTIVITY DATA

ID	GRUPO	AGE	YEARS OF FORMAL STUDY	ACR	BP-CSI	BDNF_PRE_ng_mL	BDNF_POST_ng_mL	BDNF_DELTA	NPS_PRE	NPS_POST	BDI_PRE	BDI_POST	BDI_DELTA	FIQ_PRE	FIQ_POST	FIQ_DELTA	BP-PCS_PRE	BP-PCS_POST	BP-PCS_DELTA	PSQI_PRE	PSQI_POST	PSQI_DELTA	N_SESSOES	Employed	Smoking	Drinking	ANTDEPRESANT	BENZODIAZEPINES	ANTICONVULSIVANTS	OPIOID ANALGESIC	NON-OPIOID ANALGESIC	DELTA_Freq. ins_r-acc_l	Delta_Freq. dlpfc_l-ins_r	Theta_Freq dlpfc_l-acc_l	Eyes Closed (PRE-POST) Beta-3_Freq ins_l-s1_l	Eyes Closed (PRE-POST) Beta-3_Freq ins_l-s1_r	Eyes Open (PRE-POST) Gamma_Freq ins_l-ins_r
1	1	47	17	17	34	9,6	40,84	-31,24	8,1	6,67	4	7	-3	47,19	46,309	0,88	6	4	2	7	7	0	18	1	2	2	1	2	2	2	1	-0,00519		0,00648	-0,00099097	-0,000149926	
2	1	46	9	23	57	119,18	70,06	49,12	9,9	8,33	23	28	-5	62,84	76,028	-13,19	32	29	3	4	9	-5	20	1	2	1	1	2	2	1	1	-0,00121		-0,01479	-0,001538727	-0,000667874	
3	1	52	20	21	60	25,91	19,79	6,12	7,29	7,22	11	10	1	47,53	47,339	0,19	7	6	1	9	10	-1	11	1	2	1	1	2	2	2	1	-0,00096		0,00347	-0,000646633	-0,000237022	
4	1	35	11	24	84	75,61	5,23	70,38	8,6	7,78	41	26	15	75,88	74,313	1,57	52	39	13	16	18	-2	17	1	1	2	2	1	2	1	1	-0,00087		0,01566	-0,000699737	-0,000209118	
5	1	57	6	20	69	17,51	11,14	6,37	8,6	7,78	17	13	4	78,53	62,237	16,29	42	33	9	14	11	3	11	1	2	2	1	1	2	2	1	-0,00226		0,0023	-0,000305075	-0,000242883	
6	1	42	8	23	52	31,64	32,84	-1,2	9,9	4,44	16	29	-13	76,4	72,742	3,66	38	30	8	17	11	6	10	2	1	2	2	1	2	1	1	-0,00156		-0,01001	0,000792206	0,000757242	
7	1	48	17	19	70	93,74	58,11	35,63	7,4	8,33	28	31	-3	80,84	77,417	3,42	35	29	6	15	14	1	20	1	2	2	1	2	1	1	1	-0,00278		0,01956	0,000307222	4,3913E-05	
8	1	55	11	21	62	63,65	37,14	26,51	7,3	2,22	37	15	22	76,24	63,021	13,22	42	31	11	14	4	10	20	1	2	1	2	2	2	2	1	-0,00188		0,0147	0,002186558	0,0006113642	
9	1	45	9	26	93	125,7	15,06	110,64	10	10	37	59	-22	93,73	98,667	-4,94	34	40	-6	17	14	3	20	2	2	2	1	2	1	1	1	-0,00245		0,00969	0,001639682	0,000548241	
10	1	55	11	23	65	35,47	38,7	-3,23	5,2	5,56	34	35	-1	62,41	41,744	20,67	45	33	12	7	5	2	19	2	2	2	2	2	2	2	1	-0,00545		0,02093	-4,72970000000004E-05	-0,000328508	
11	1	30	17	22	52	4,67	48,59	-43,92	8,17	7,22	15	11	4	61,93	59,645	2,28	29	31	-2	13	11	2	19	2	2	2	1	1	2	1	1	0,00209		-0,0061	-0,00017882	-5,3146E-05	
12	1	57	11	32	83	15,31	24,22	-8,91	9,6	8,89	39	24	15	79,95	82,169	-2,22	51	47	4	18	14	4	20	1	1	2	1	2	2	1	1	-0,00073		-0,00303	0,000145207	4,50519999999999E-05	
13	1	42	11	25	53	23,95	49,92	-25,97	9,32	5,56	20	18	2	81,54	66,299	15,24	32	13	19	11	6	5	15	1	2	2	2	2	2	2	1	5,99999999999976E-05		0,00458	0,000520477	0,000101614	
14	1	46	13	23	80	31,33	53,1	-21,77	8,94	4,44	10	2	8	70,63	30,076	40,55	36	10	26	11	9	2	19	1	1	2	1	2	2	2	1	-0,00419		0,01442	-0,000895409	-0,000140991	
15	1	64	11	25	47	30,17	31,68	-1,51	7,7	3,89	13	3	10	25,01	35,085	-10,08	25	12	13	11	13	-2	20	1	2	2	1	2	2	2	1	-0,00178		-0,0067	1,72040000000003E-05	-1,63990000000001E-05	
16	1	59	10	30	79	123,24	18,49	104,75	9,6	8,89	48	40	8	76,64	91,253	-14,61	42	43	-1	15	14	1	12	2	2	2	1	2	1	2	1	0,00075		-0,00621	-0,000156604	-2,59216E-05	
17	2	62	6	24	59	14,64	14,64	0	7,1	6,67	38	23	15	67,35	53,62	13,73	38	25	13	13	6	7	20	2	2	2	2	2	2	2	1	-0,00043					
18	2	40	9	26	65	44,79	20,93	23,86	9,5	5,56	26	17	9	71,05	68,569	2,48	19	5	14	6	6	0	14	1	1	2	2	1	2	2	1	0,00251					
19	2	49	20	24	76	10,51	10,51	0	9,13	8,89	26	23	3	73,56	74,727	-1,17	40	29	11	15	13	2	18	1	2	2	1	2	1	2	1	0,00481					
20	2	35	13	20	58	19,32	32,42	-13,1	9,7	9,44	20	14	6	47,15	82,027	-34,88	52	51	1	14	13	1	14	1	2	2	1	2	2	1	1	0,00074					
21	2	44	12	26	66	34,17	27,59	6,58	9,2	6,67	39	21	18	77,14	81,932	-4,79	42	34	8	18	18	0	17	1	2	2	1	1	1	1	1	-0,00081					
22	2	60	12	21	52	87,08	172,52	-85,44	9,1	0,56	14	12	2	78,28	26,522	51,76	44	18	26	6	8	-2	20	2	1	2	1	2	2	2	1	0,00215					
23	2	35	11	31	75	33,67	46,84	-13,17	8,46	8,33	31	24	7	69,89	61,681	8,21	42	28	14	14	7	7	17	1	2	2	1	2	1	2	1	0,00114					
24	2	42	17	24	67	9,23	58,22	-48,99	7,1	5,56	32	17	15	73,84	56,677	17,16	32	24	8	8	4	4	20	2	2	2	2	2	2	2	1	0,00139					
25	3	54	10	24	45	72,55	36,3	36,25	6,25	7,78	9	6	3	56,82	43,201	13,62	22	9	13	13	14	-1	20	1	2	2	2	2	2	2	1		0,00066918				
26	3	55	11	27	72	20,34	63,48	-43,14	8,64	5,56	27	22	5		70,823	-2	41	43	1	13	12	5	26	2	1	2	1	2	2	2	1		0,0001619				
27	3	65	14	19	58	43,23	37,45	5,78	10	8,89	10	8	2	69,68	25,606	44,07	31	24	7	12	10	2	20	1	2	2	2	2	2	2	1		0,00065606				
28	3	30	11	26	77	25,32	26,57	-1,25	9,6	8,89	24	19	5	83,83	73,581	10,25	45	44	1	9	7	2	20	2	2	2	1	2	2	2	1		0,00055899				
29	3	51	15	24	77	28,34	27,53	0,81	9,7		40			94,65	93,79	0,86	36	45	-9	18	20	-2	13	2	1	2	1	2	1	1	1		-0,00105446				
30	3	37	10	21	39	38,07	46,25	-8,18	8,1		27	30	-3	42,92	70,23	-27,31	42	40	2	13	18	-5	8	1	2	2	2	2	2	2	1		-0,00053209				
31	3	53	12		51	60,04	40,43	19,61	5	9,44	17	9	8	38,29	62,747	-24,46	30	38	-8	12	9	3	23	1	2	1	1	2	2	2	1		-0,0024794				
32	3	39	18	27	69	17,9	32,49	-14,59	7,21	4,44	19	2	17	67,51	49,443	18,07	33	16	17	9	11	-2	19	2	2	1	1	2	2	2	1		0,00278317				
33	3	56	5	23	77	28,14	10,84	17,3	9	5	19	3	16	78,96	45,25	33,71	34	38	-4	10	6	4	24	1	1	2	2	2	2	2	1		-0,00015621				
34	3	62	13	25	77	23,24	56,99	-33,75	7	6,67	22	6	16	66,26	65,88	0,38	49	42	7	14	10	4	20	1	2	2	1	2	1	1	1		-0,00036232				
35	3	42	11	19	76	56,69	27,91	28,78	8	7,78	35	29	6	78,68	63,4	15,28	45	41	4	13	14	-1	20	2	1	2	2	2	2	2	2		-0,00023149				
36	3	63	5	24	36	64,76	38,1	26,66	10	6,67	31	26	5	82,08	61,2	20,88	48	35	13	19	16	3	21	2	2	1	1	2	2	2	2		0,00225954				
37	3	35	16	23	64	30,67	45,66	-14,99	8	7,78	12	19	-7	67,68	69,2	-1,52	41	46	-5	8	16	-8	11	1	2	2	1	2	2	2	2		-0,00249855				
38	4	42	8	19	67	23	24,22	-1,22	8	5,56	32	30	2	74,96	63,871	11,09	27	29	-2	13	14	-1	20	2	2	1	1	2	1	1	1			0,00981			-6,22760000000001E-05
39	4	34	16	22	62	18,11	26,76	-8,65	6,7	7,78	13	9	4	58,28	59,62	-1,34	19	17	2	11	11	0	14	1	2	2	1	1	1	2	1			-0,03142			-2,7693E-05
40	4	43	16	17	52	85,38	12,62	72,76	4,3	3,33	20	9	11	42,06	21,842	20,22	42	40	2	9	11	-2	14	2	1	1	1	2	2	2	1			-0,02805			-5,1867E-05
41	4	36	17	21	58	90,16	30,17	59,99	7,7	7,78	6	4	2	64,75	53,046	11,7	18	16	2	10	6	4	19	1	2	2	1	2	2	1	1			-0,01286			-6,4929E-05
42	4	55	13	19	41	24,56	14,81	9,75	8,1	7,22	12	8	4	22,67	57,014	-34,34	29	16	13	11	13	-2	20	1	2	2	2	2	2	2	1			-0,02603			-3,7366E-05
43	4	55	13	27	60	4,67	44,39	-39,72	8	6,67	15	30	-15	70,84	57,683	13,16	28	14	14	10	8	2	13	2	2	2	1	2	1	2	1			-0,02386			-2,8634E-05
